# Supplementary material for: SIRT2 inhibition protects against cardiac hypertrophy and ischemic injury
Source: eLife. 2023 Sep 20;12:e85571. doi: 10.7554/eLife.85571 (PMC10558204; doi:10.7554/eLife.85571)
Supplement: Figure 1—source data 4. [file elife-85571-fig1-data4.pptx]

## Slide 1
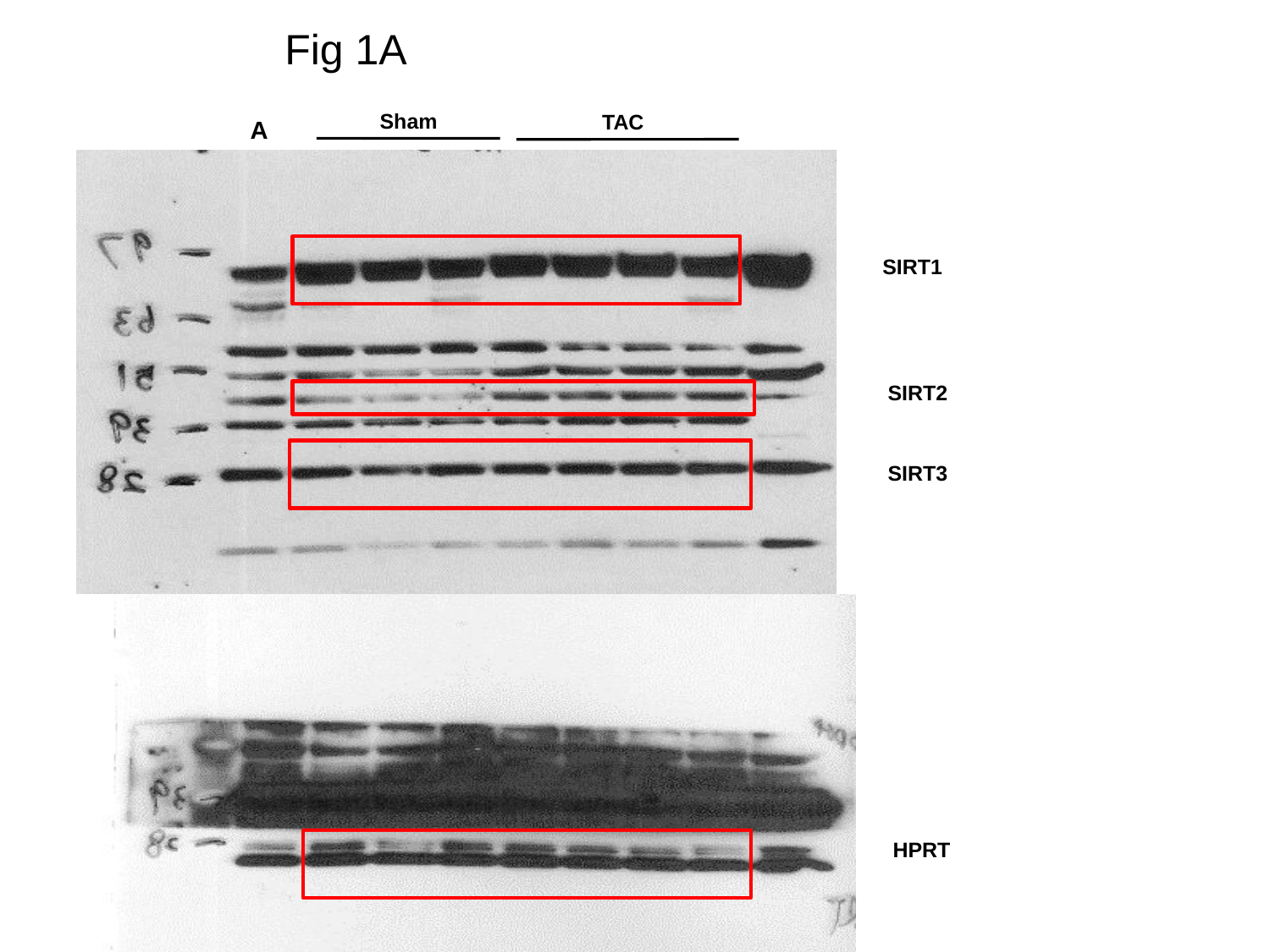

Fig 1A
Sham
TAC
A
SIRT1
SIRT2
SIRT3
HPRT

## Slide 2
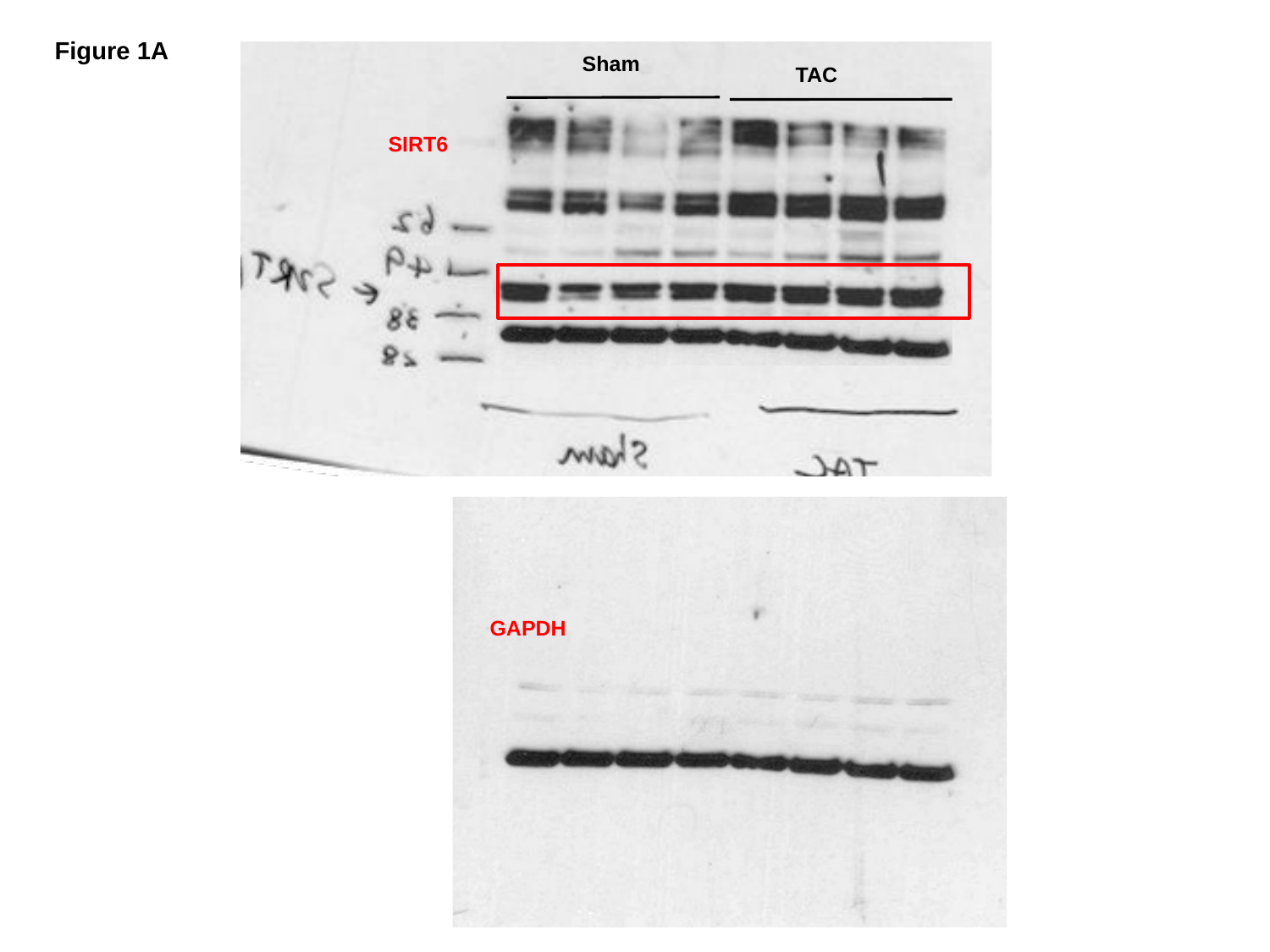

Figure 1A
Sham
TAC
SIRT6
GAPDH

## Slide 3
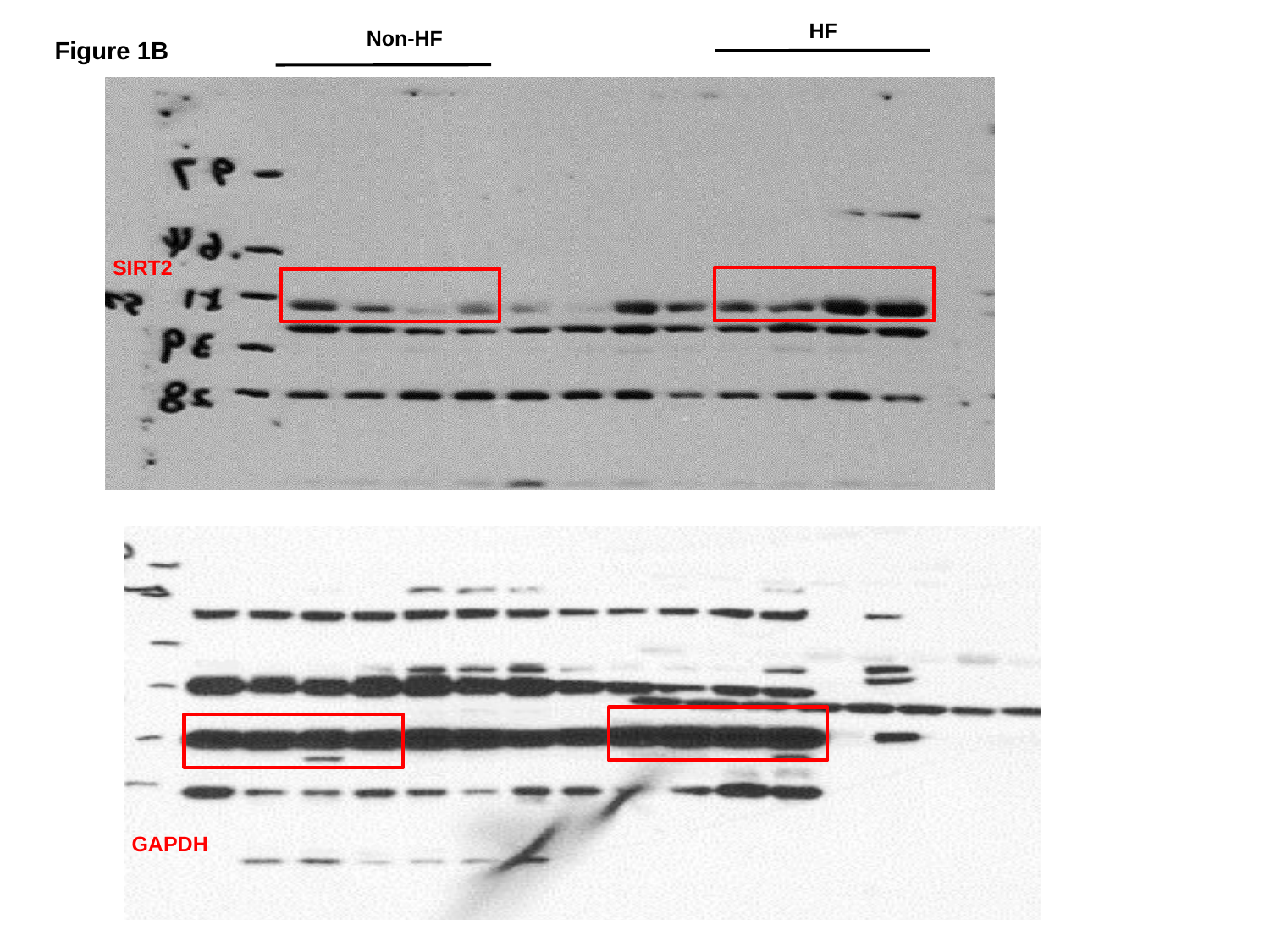

HF
Non-HF
Figure 1B
SIRT2
GAPDH

## Slide 4
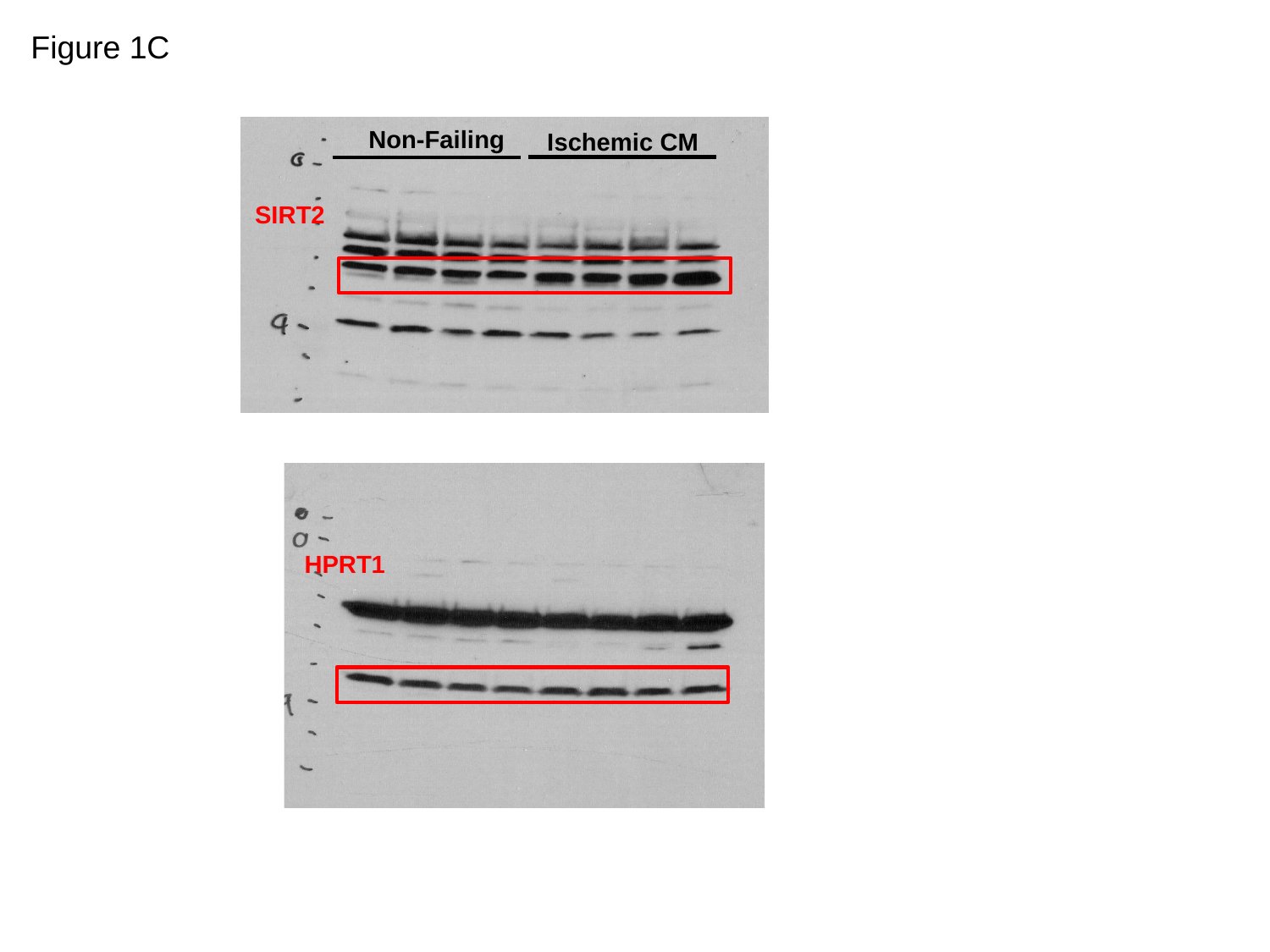

Figure 1C
SIRT2
Non-Failing
Ischemic CM
HPRT1
