# Supplementary material for: SIRT2 inhibition protects against cardiac hypertrophy and ischemic injury
Source: eLife. 2023 Sep 20;12:e85571. doi: 10.7554/eLife.85571 (PMC10558204; doi:10.7554/eLife.85571)
Supplement: Figure 1—source data 5. [file elife-85571-fig1-data5.pptx]

## Slide 1
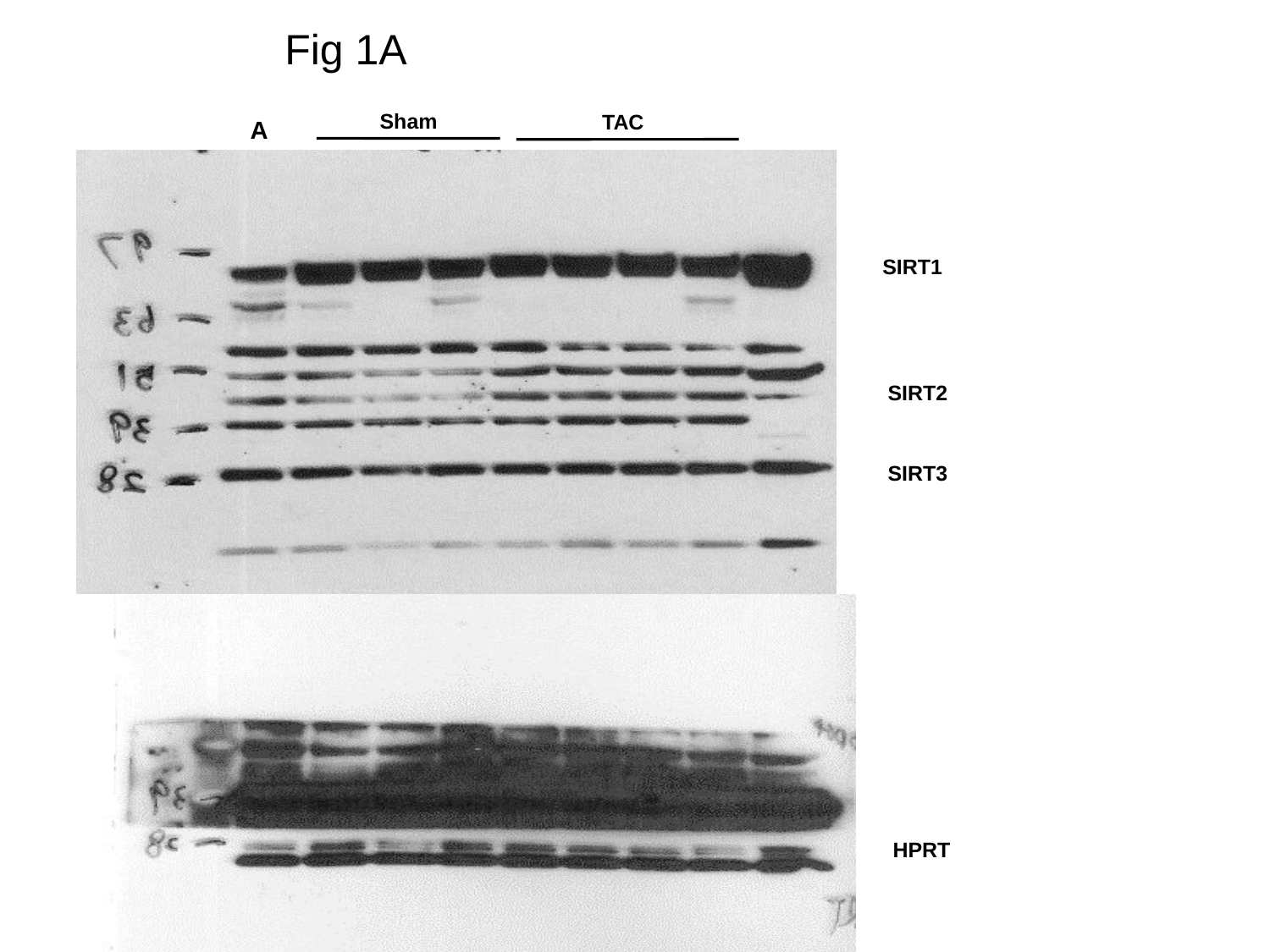

Fig 1A
Sham
TAC
A
SIRT1
SIRT2
SIRT3
HPRT

## Slide 2
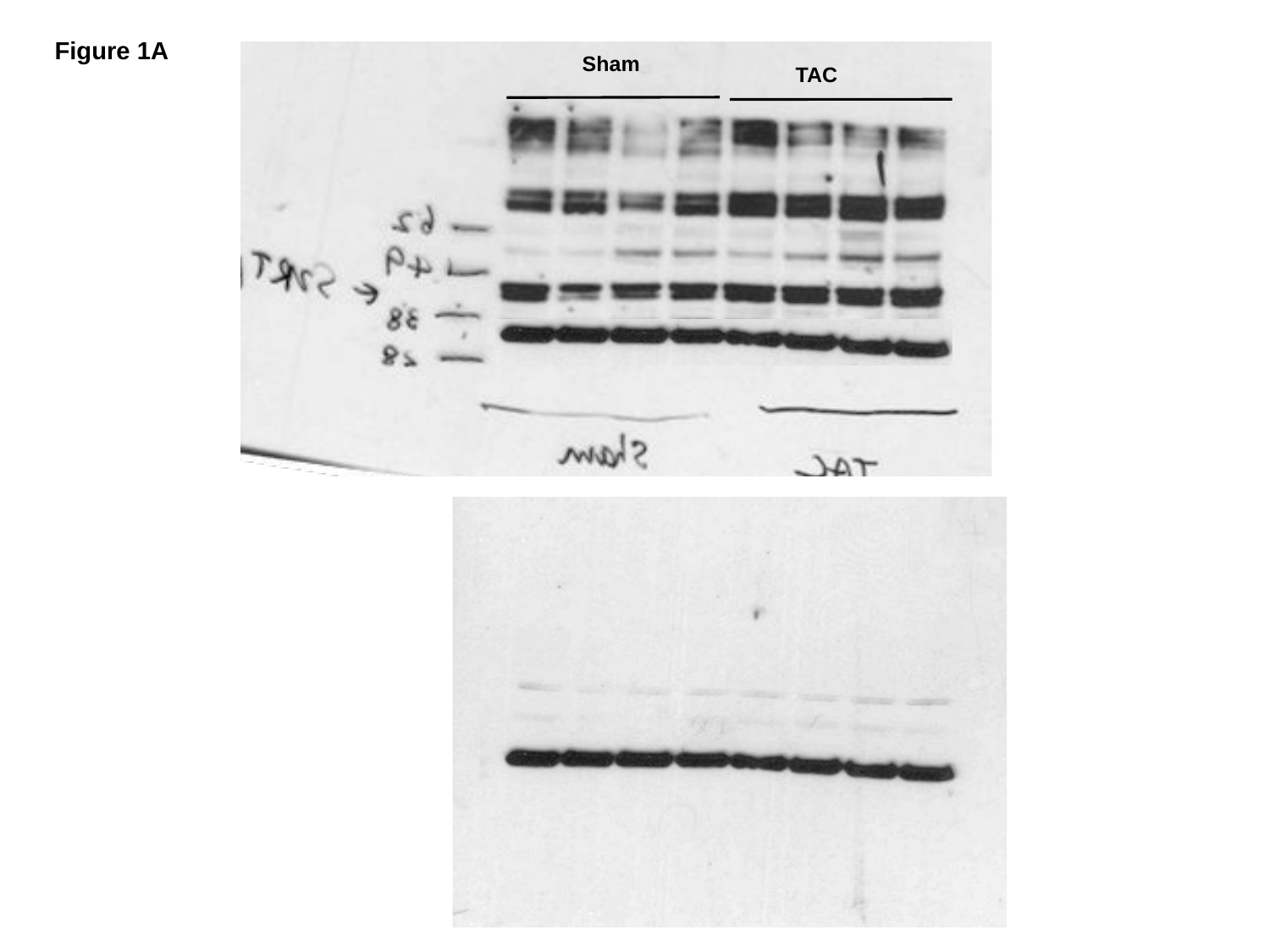

Figure 1A
Sham
TAC

## Slide 3
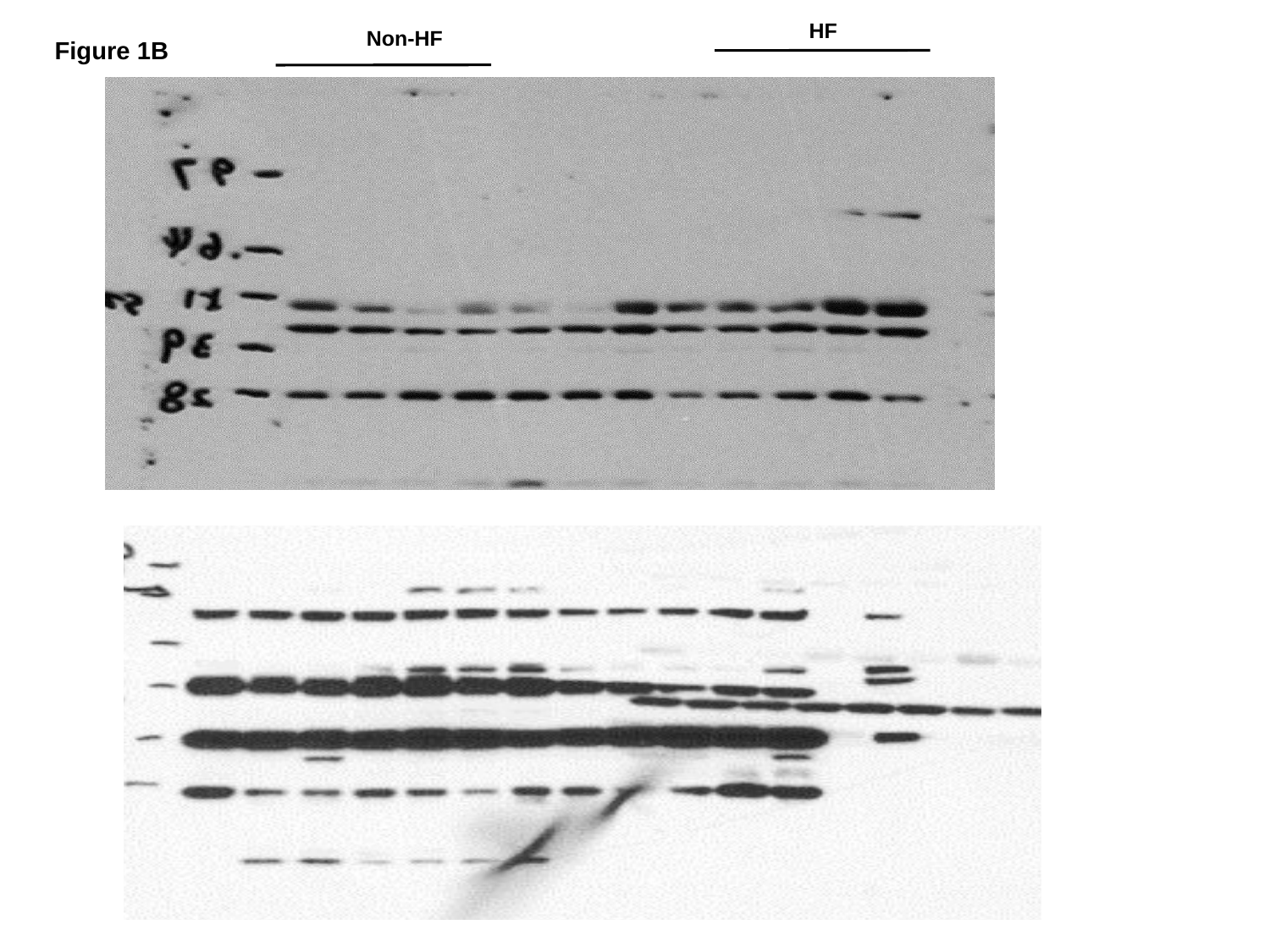

HF
Non-HF
Figure 1B

## Slide 4
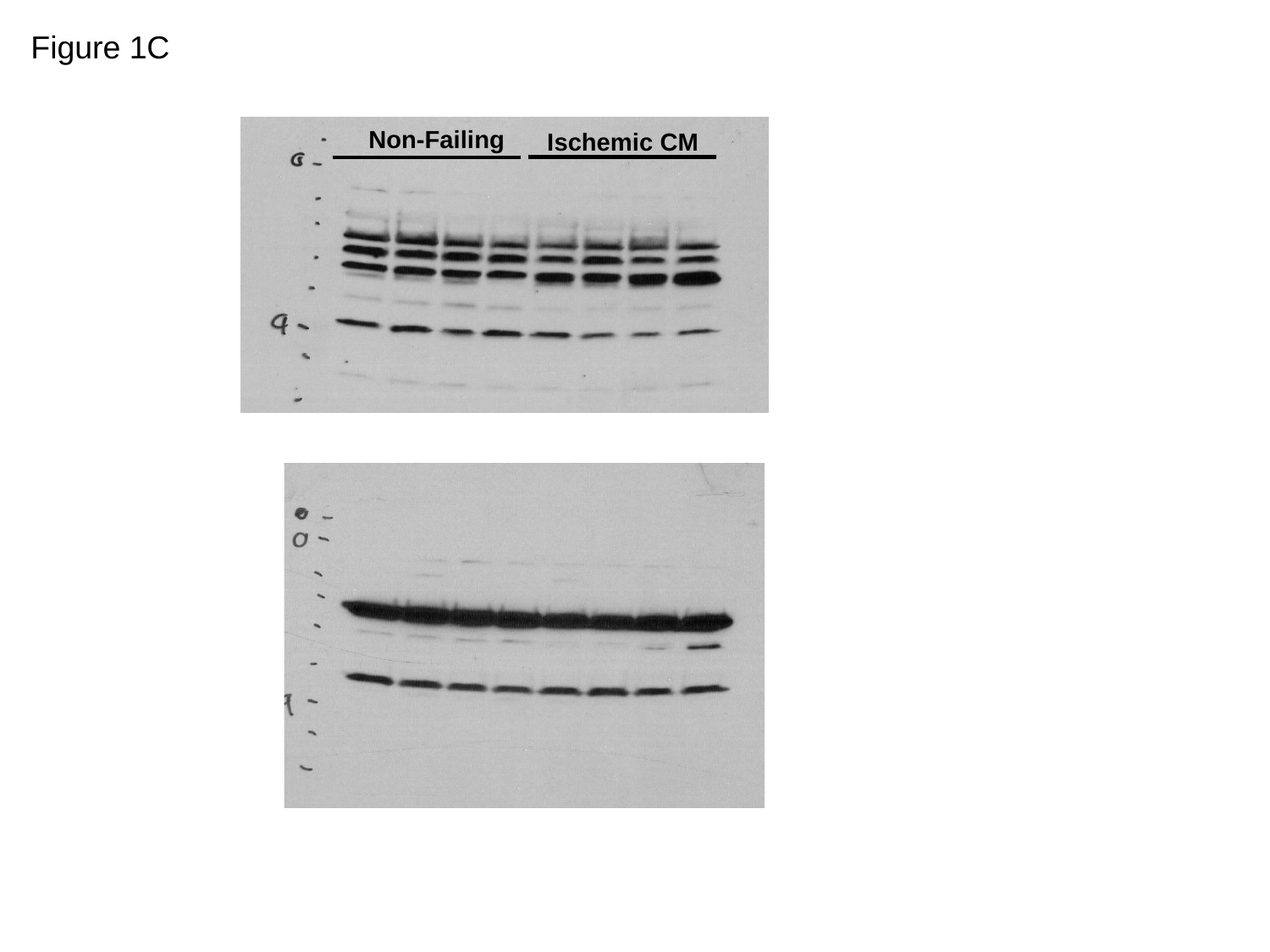

Figure 1C
Non-Failing
Ischemic CM
