# Supplementary figures and images for: SIRT2 inhibition protects against cardiac hypertrophy and ischemic injury
Source: eLife. 2023 Sep 20;12:e85571. doi: 10.7554/eLife.85571 (PMC10558204; doi:10.7554/eLife.85571)

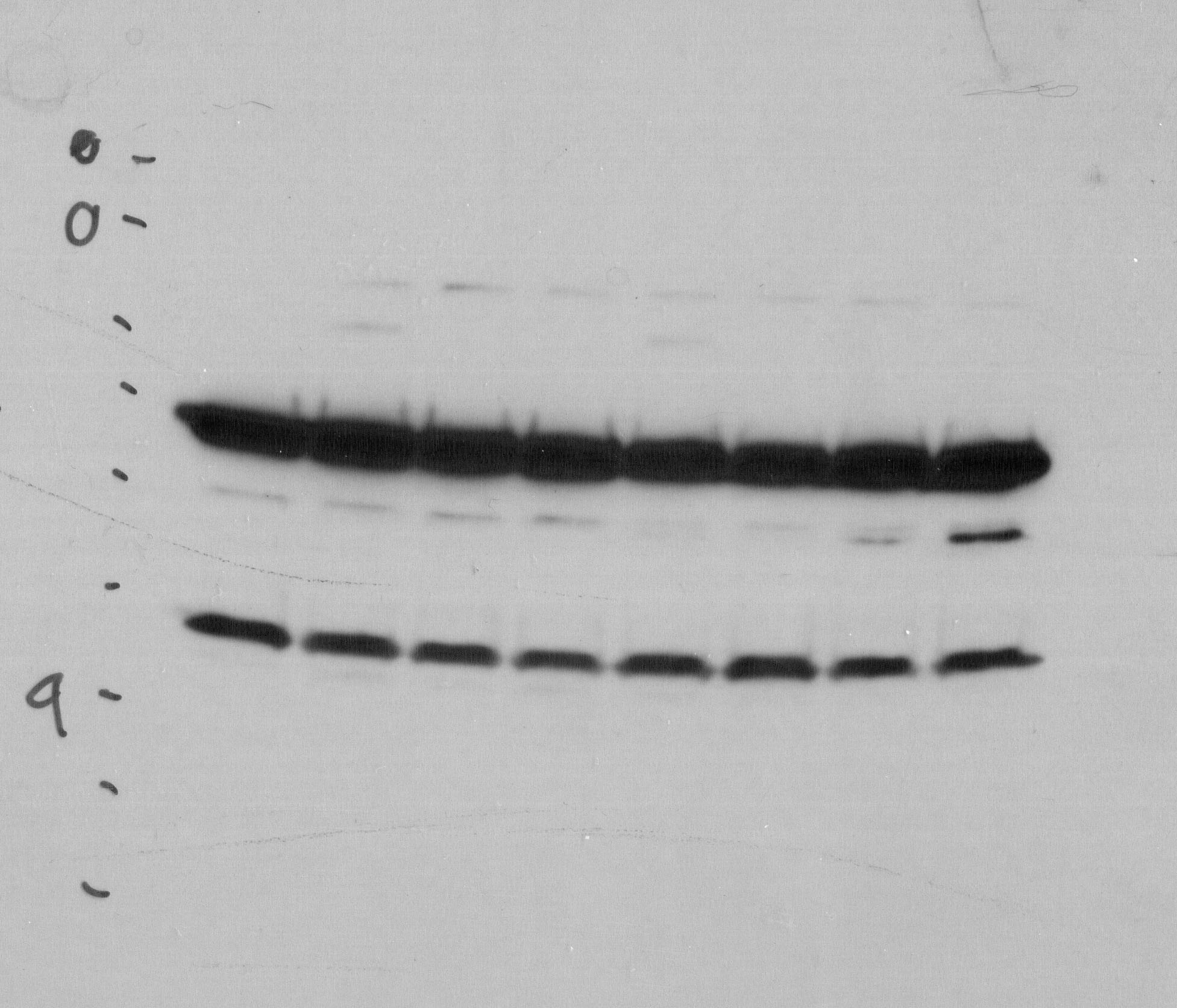

Supplement: Figure 1—source data 6. [file elife-85571-fig1-data6.zip › Fig 1C HPRT.jpg]

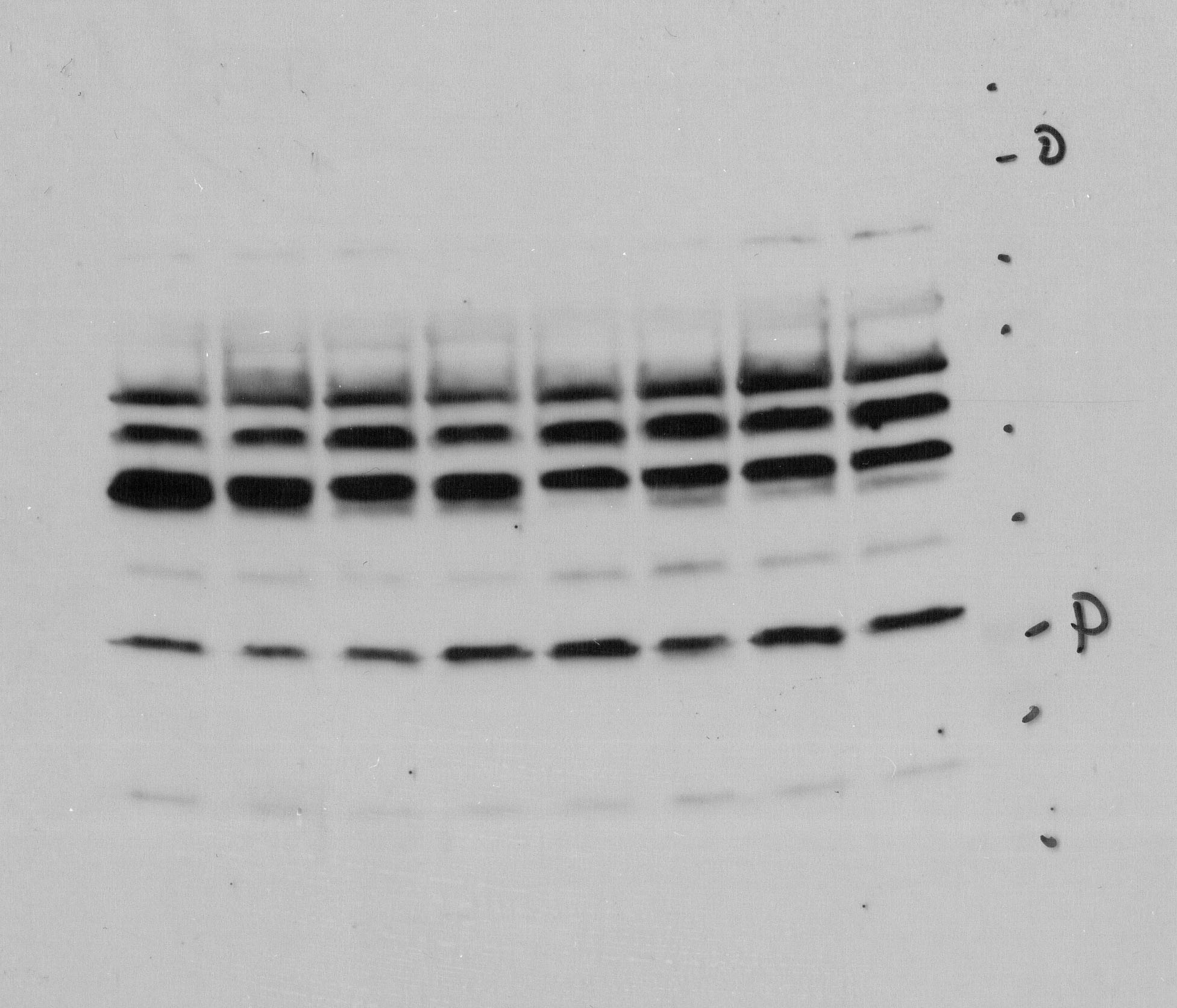

Supplement: Figure 1—source data 6. [file elife-85571-fig1-data6.zip › Fig 1C Sirt2.jpg]

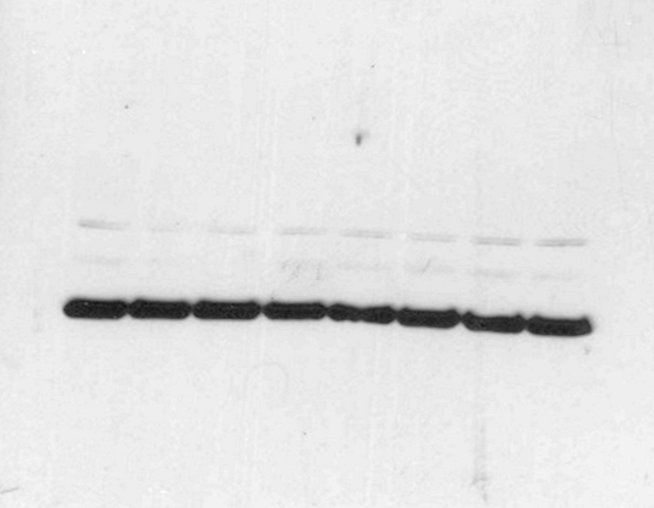

Supplement: Figure 1—source data 6. [file elife-85571-fig1-data6.zip › Figure 1A GAPDH.tif]

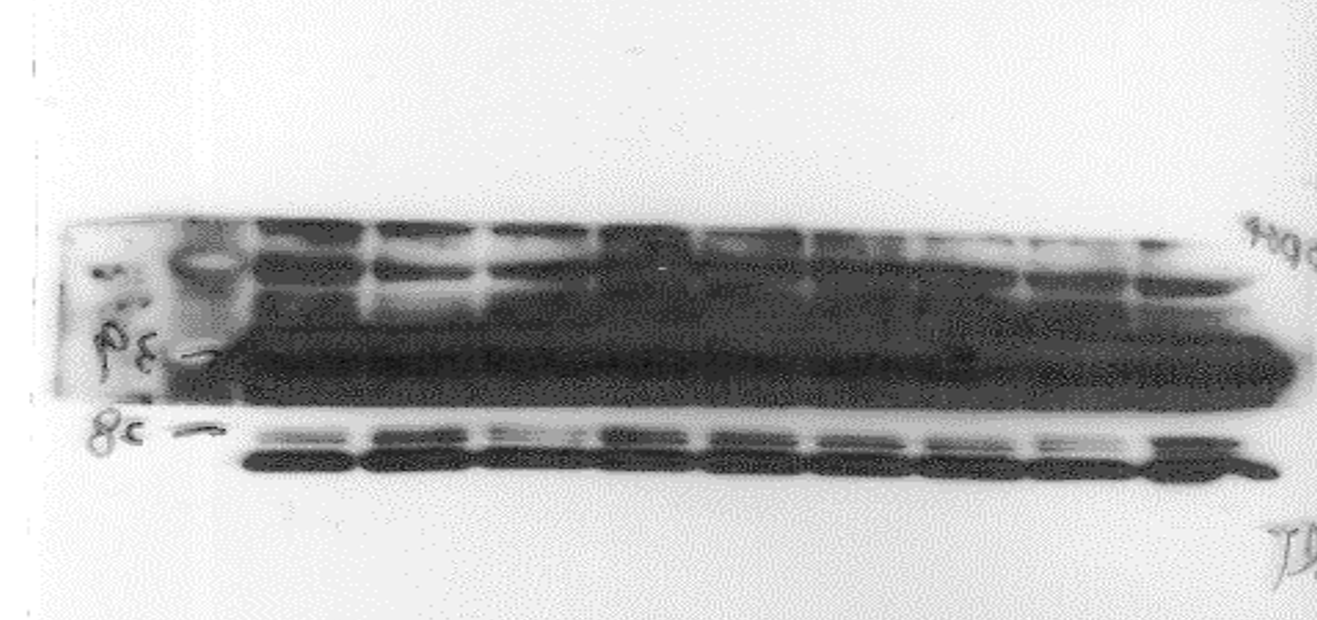

Supplement: Figure 1—source data 6. [file elife-85571-fig1-data6.zip › Figure 1A HPRT.tif]

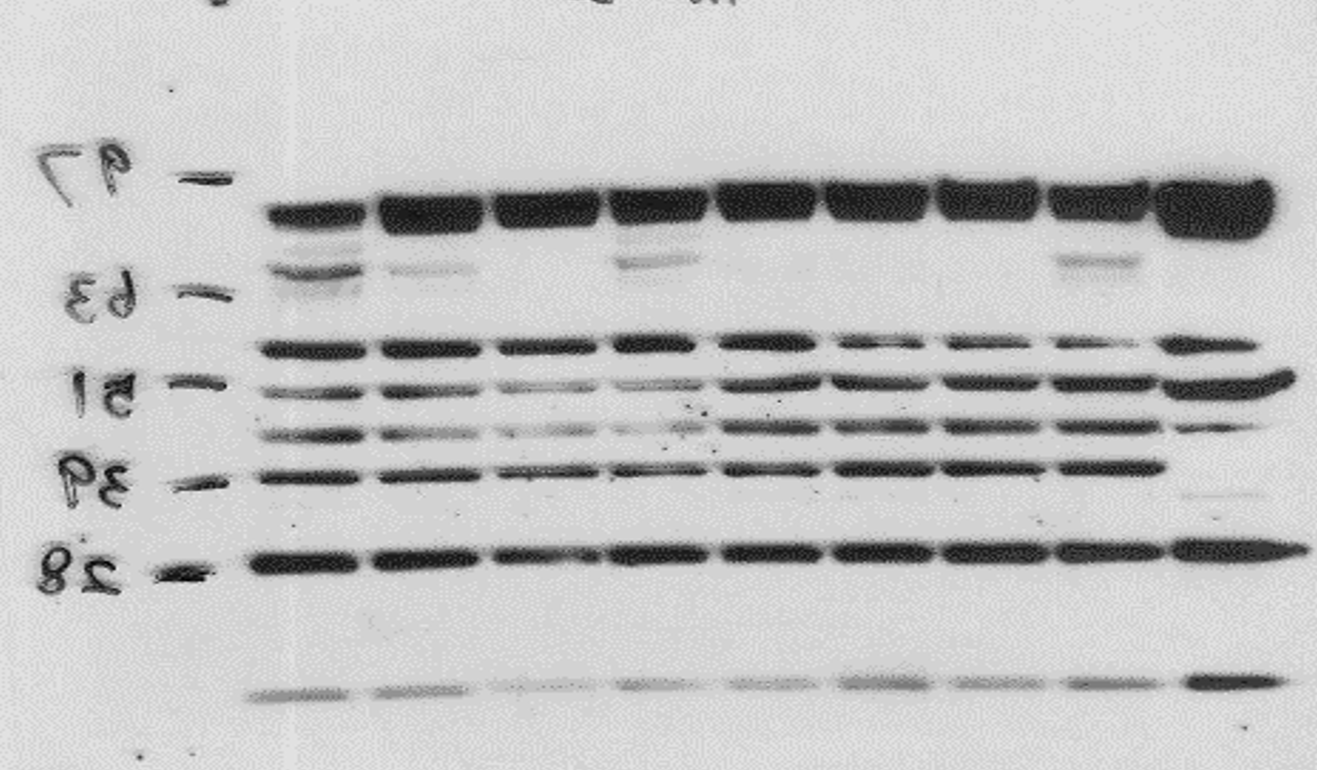

Supplement: Figure 1—source data 6. [file elife-85571-fig1-data6.zip › Figure 1A SIRT1 SIRT2 SIRT3.tif]

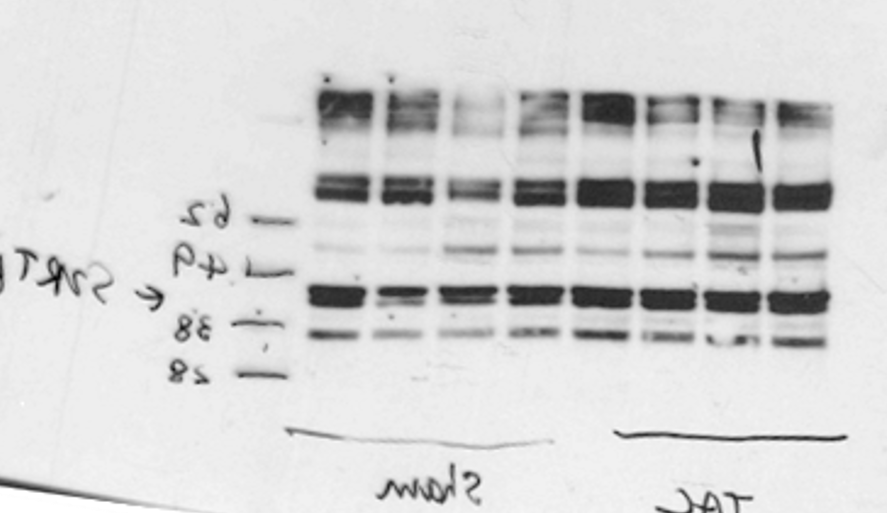

Supplement: Figure 1—source data 6. [file elife-85571-fig1-data6.zip › Figure 1A SIRT6.tif]

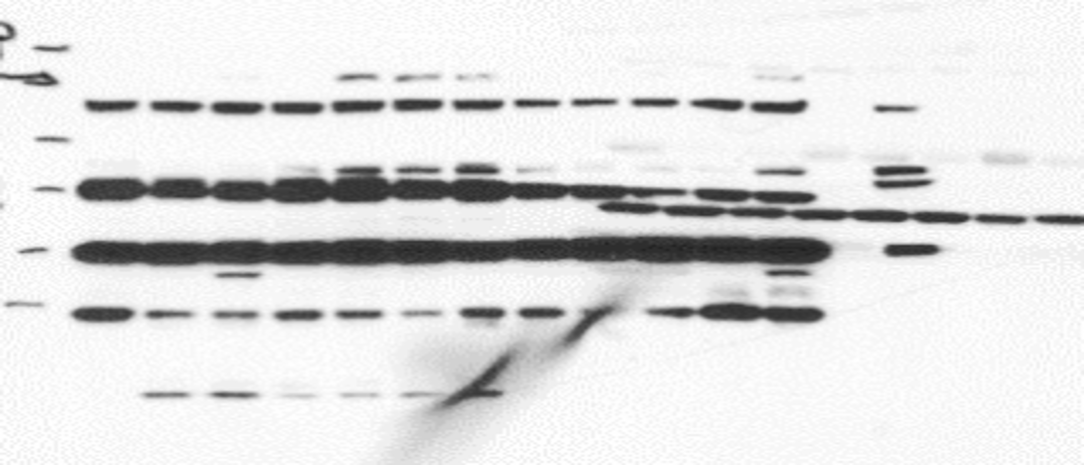

Supplement: Figure 1—source data 6. [file elife-85571-fig1-data6.zip › Figure 1B GAPDH.tif]

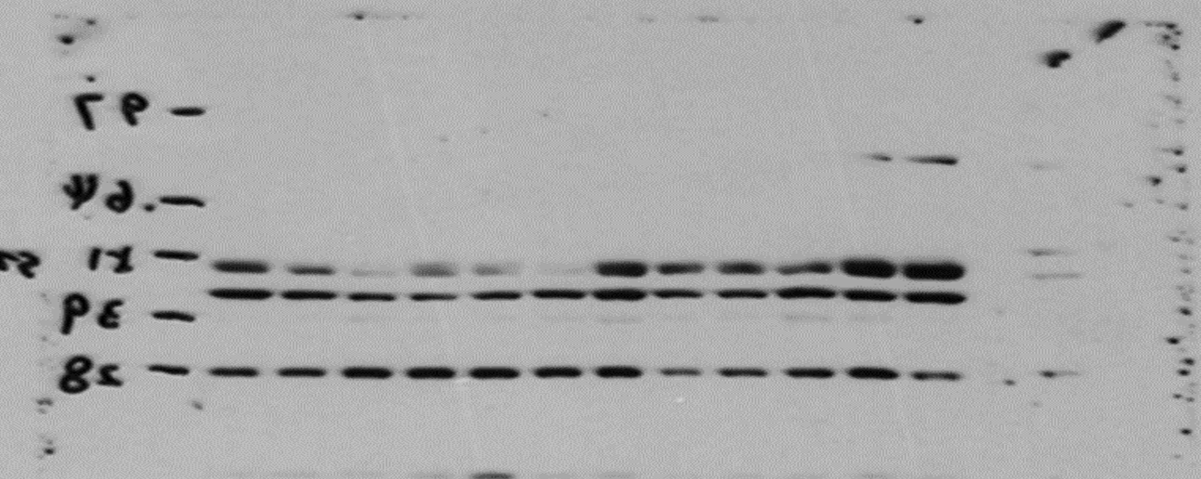

Supplement: Figure 1—source data 6. [file elife-85571-fig1-data6.zip › Figure 1B SIRT2.tif]

## Slide 1
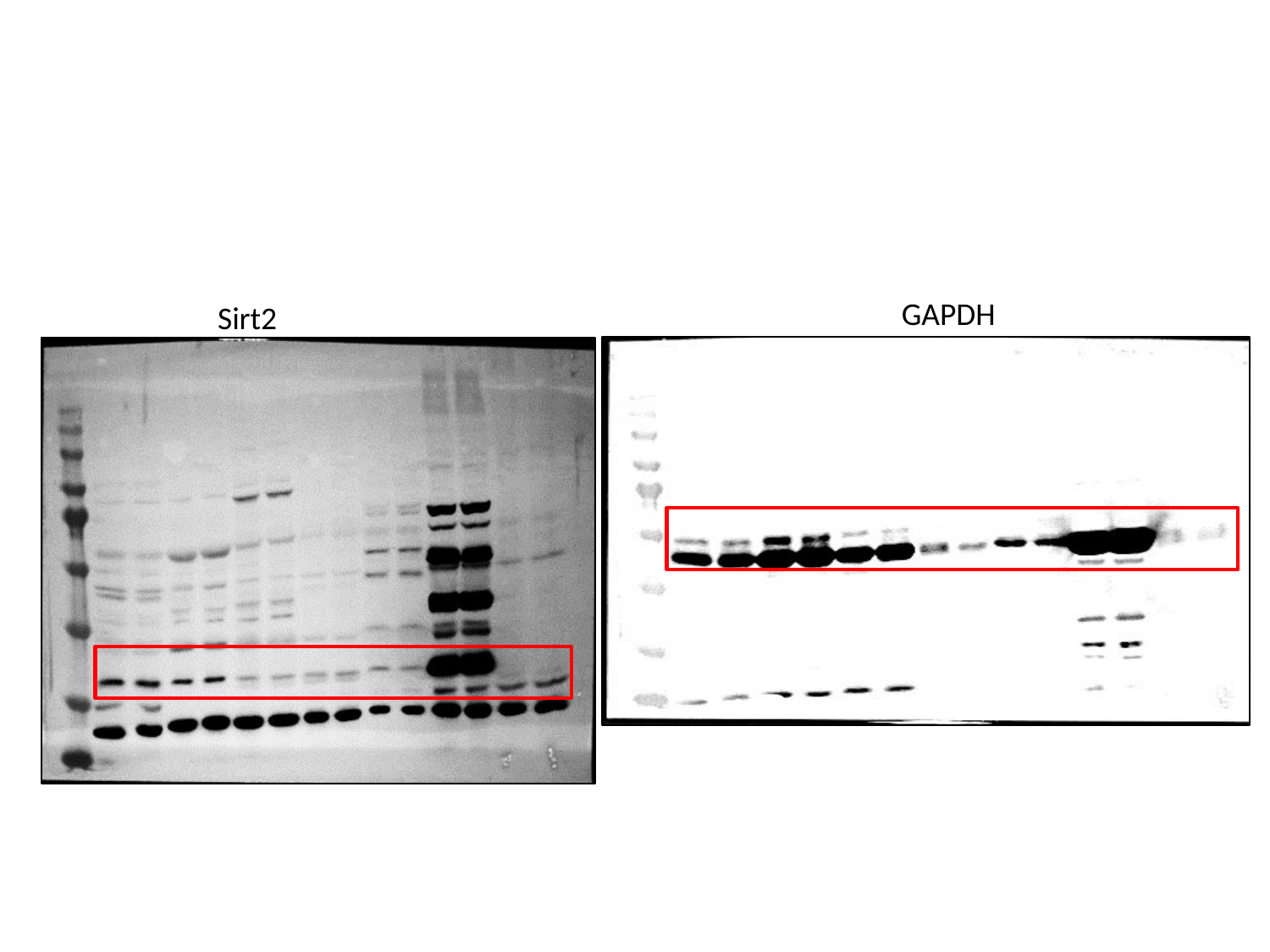

GAPDH
Sirt2

## Slide 2
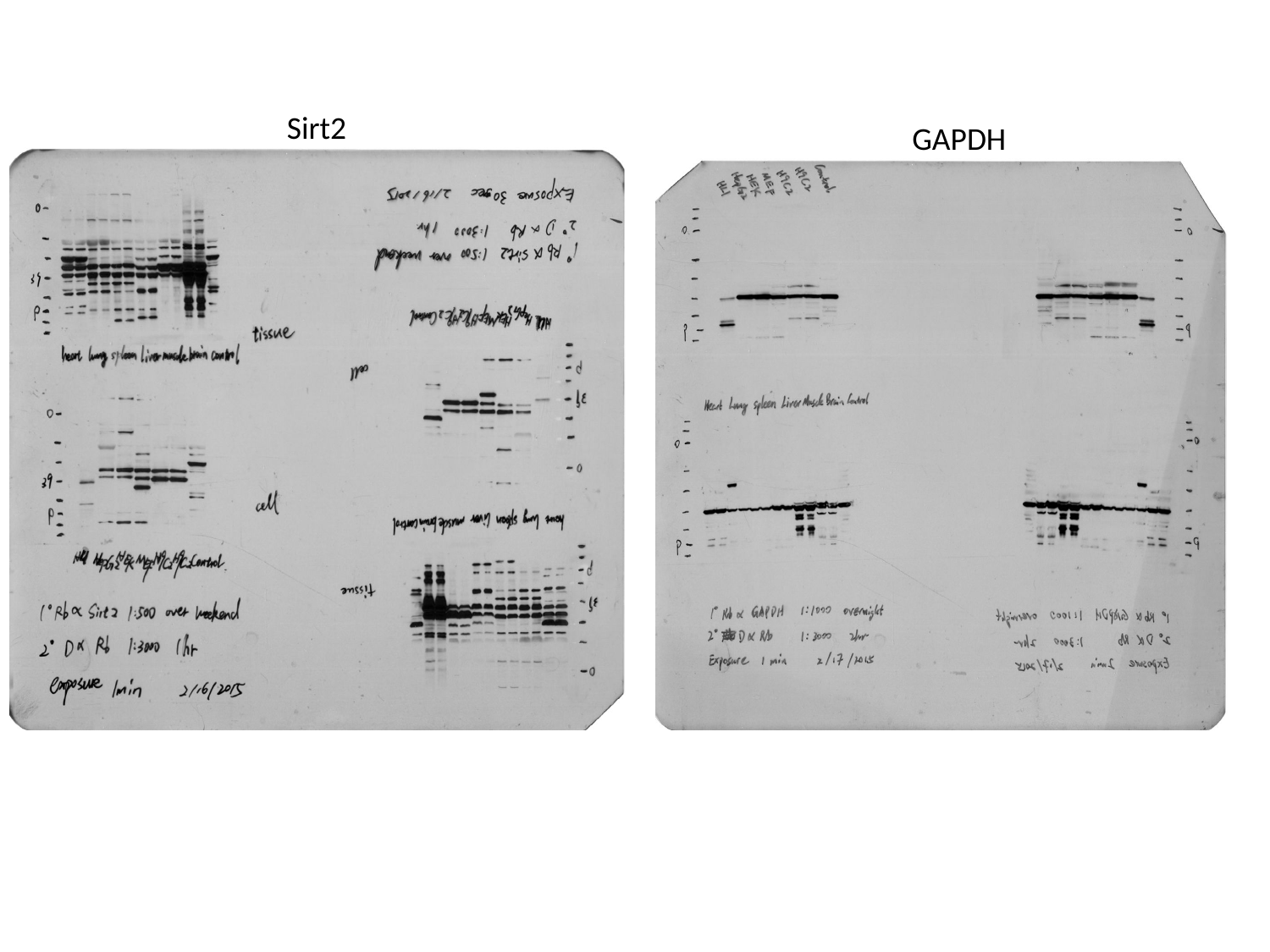

Sirt2
GAPDH

Supplement: Figure 1—figure supplement 1—source data 1. [file elife-85571-fig1-figsupp1-data1.pptx]

## Slide 1
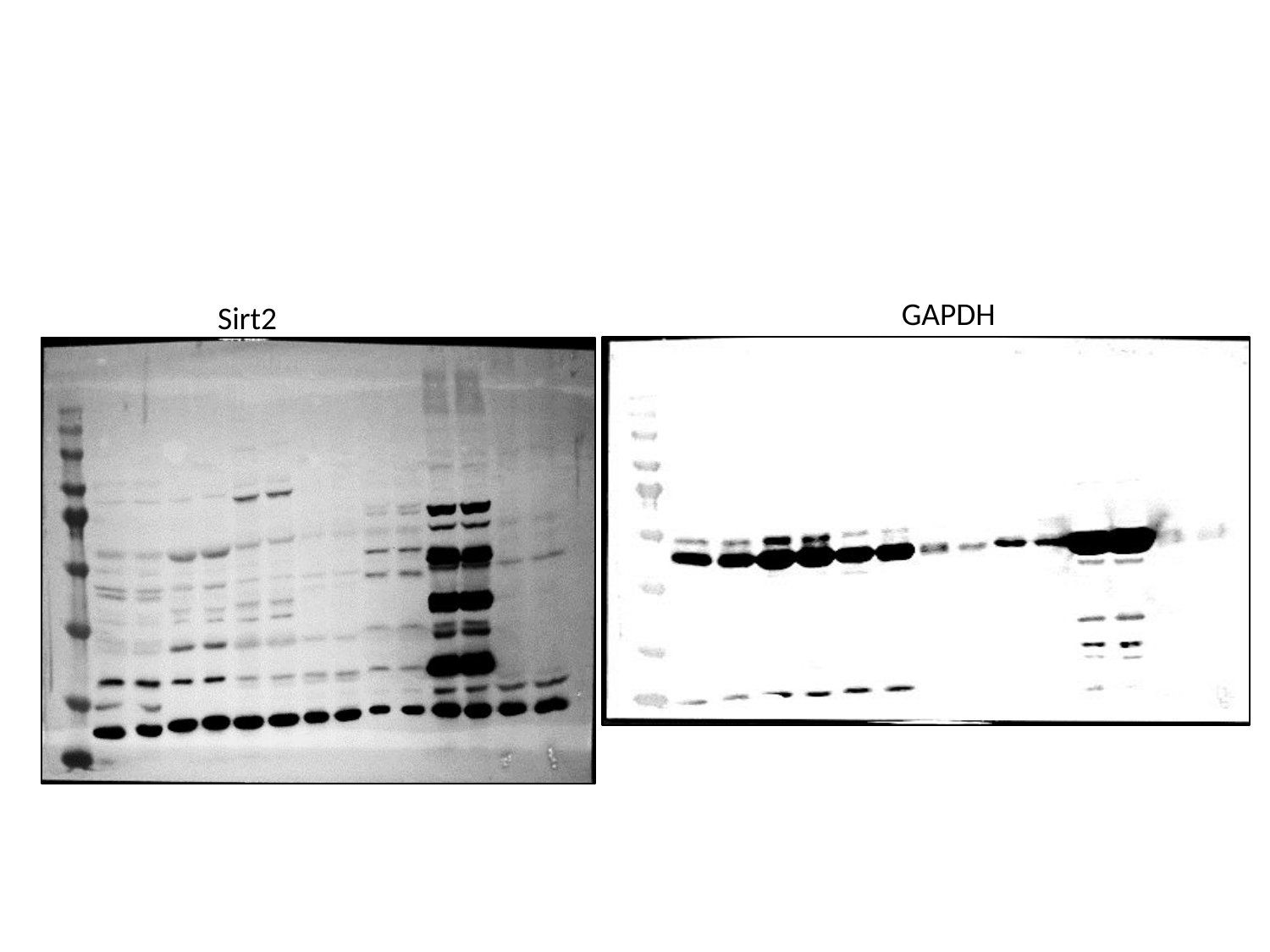

GAPDH
Sirt2

## Slide 2
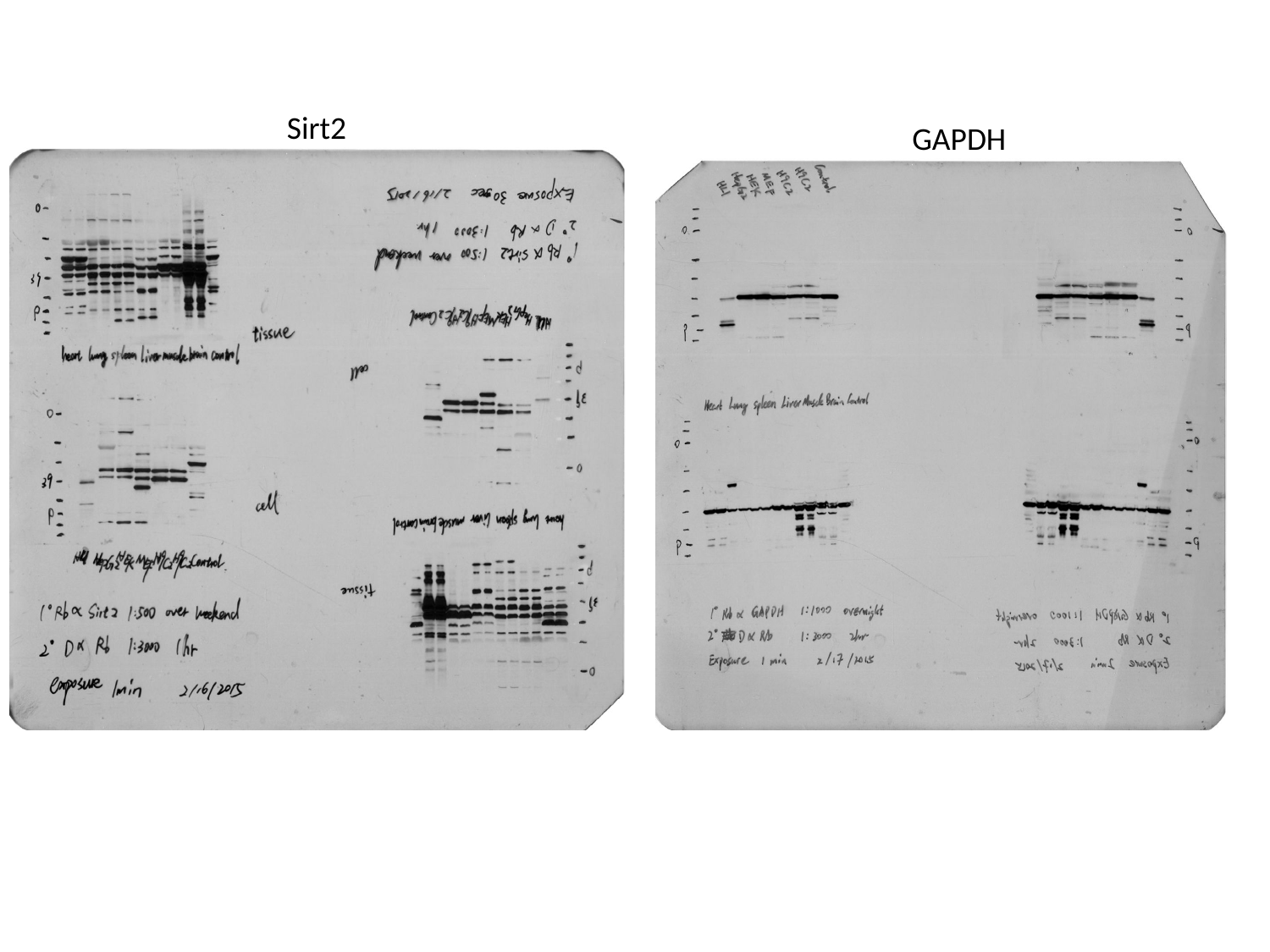

Sirt2
GAPDH

Supplement: Figure 1—figure supplement 1—source data 2. [file elife-85571-fig1-figsupp1-data2.pptx]

## Slide 1
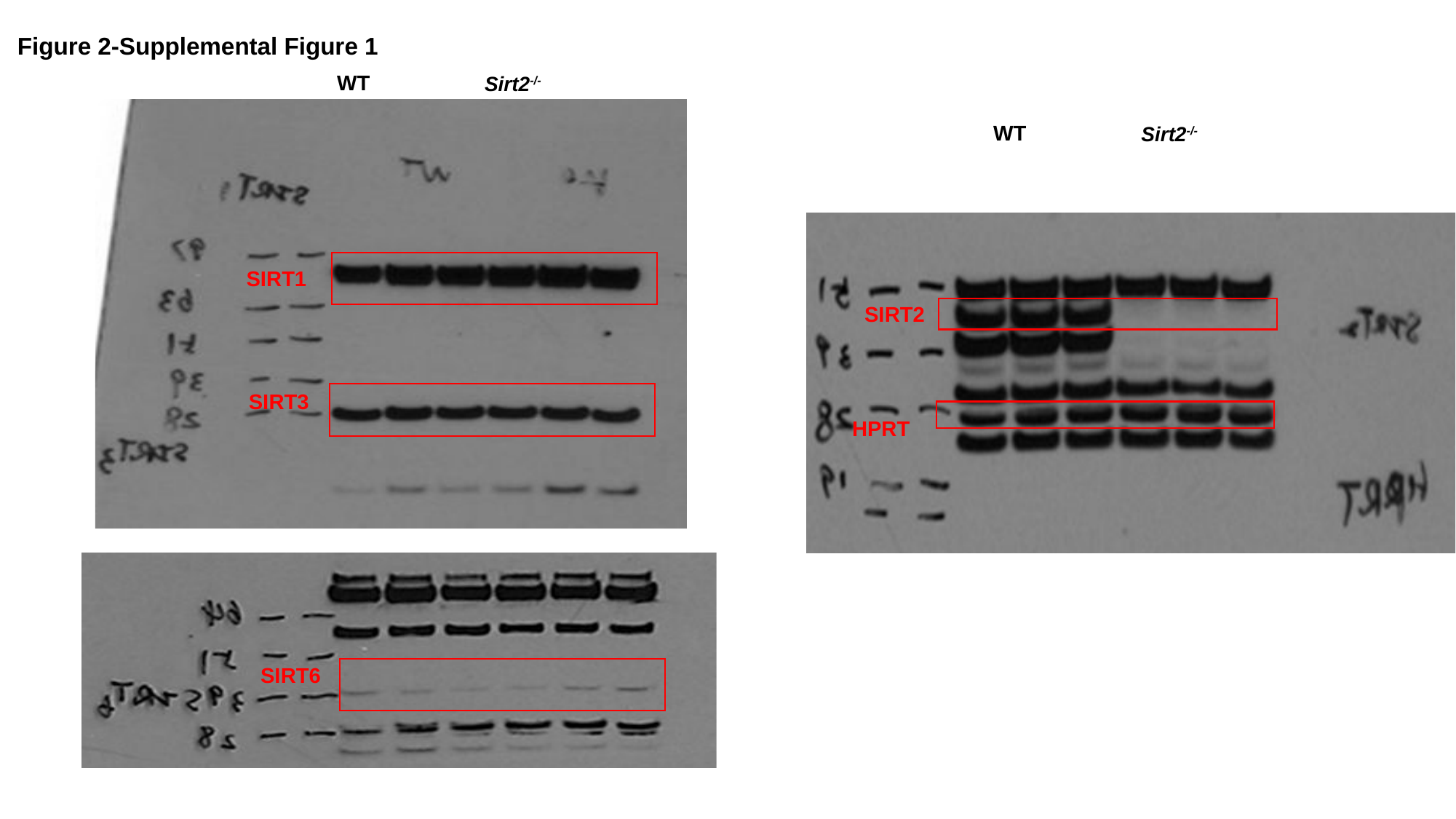

Figure 2-Supplemental Figure 1
WT
Sirt2-/-
SIRT1
SIRT3
WT
Sirt2-/-
SIRT2
HPRT
SIRT6

Supplement: Figure 2—figure supplement 1—source data 2. [file elife-85571-fig2-figsupp1-data2.pptx]

## Slide 1
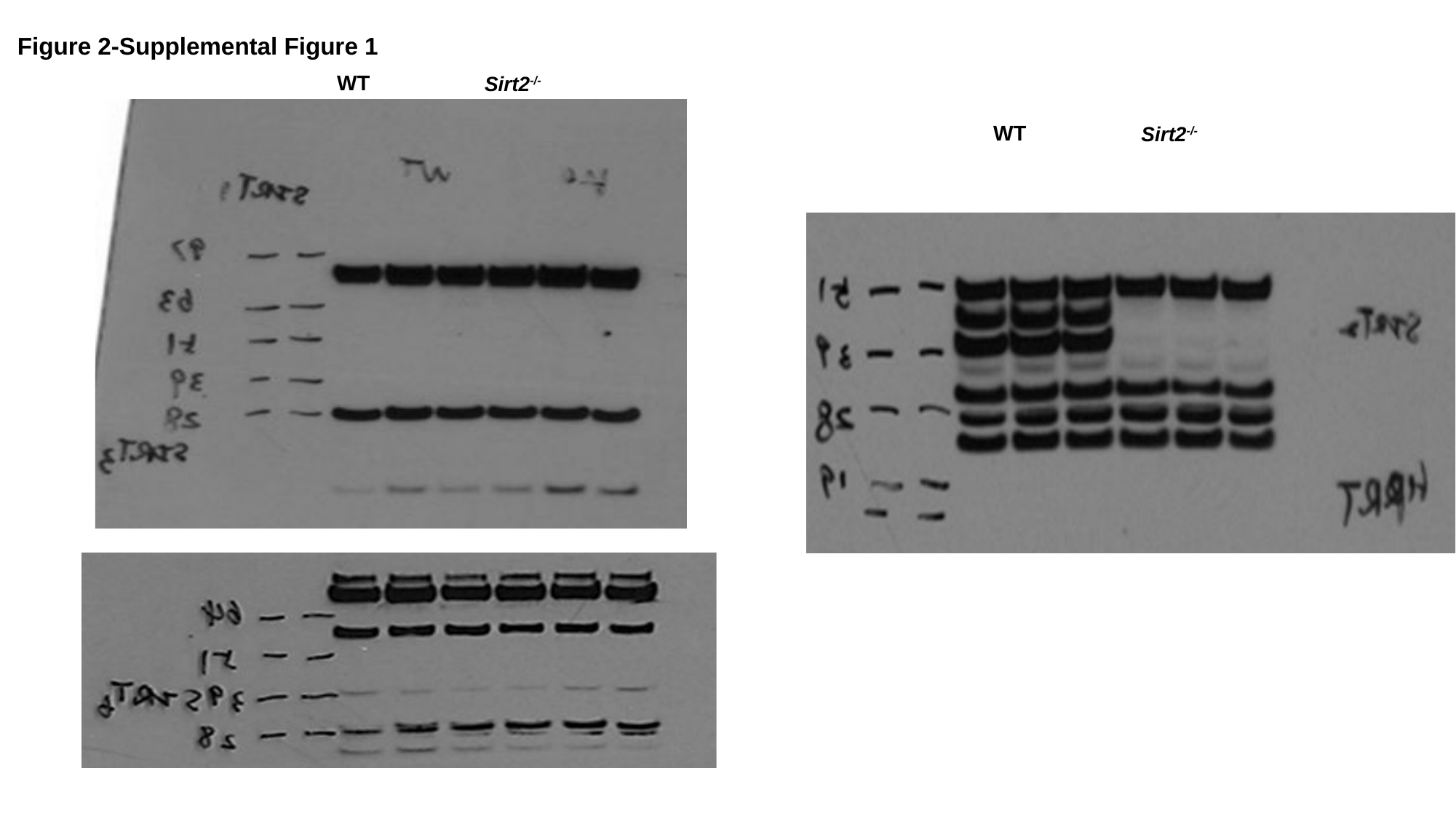

Figure 2-Supplemental Figure 1
WT
Sirt2-/-
WT
Sirt2-/-

Supplement: Figure 2—figure supplement 1—source data 3. [file elife-85571-fig2-figsupp1-data3.pptx]

## Slide 1
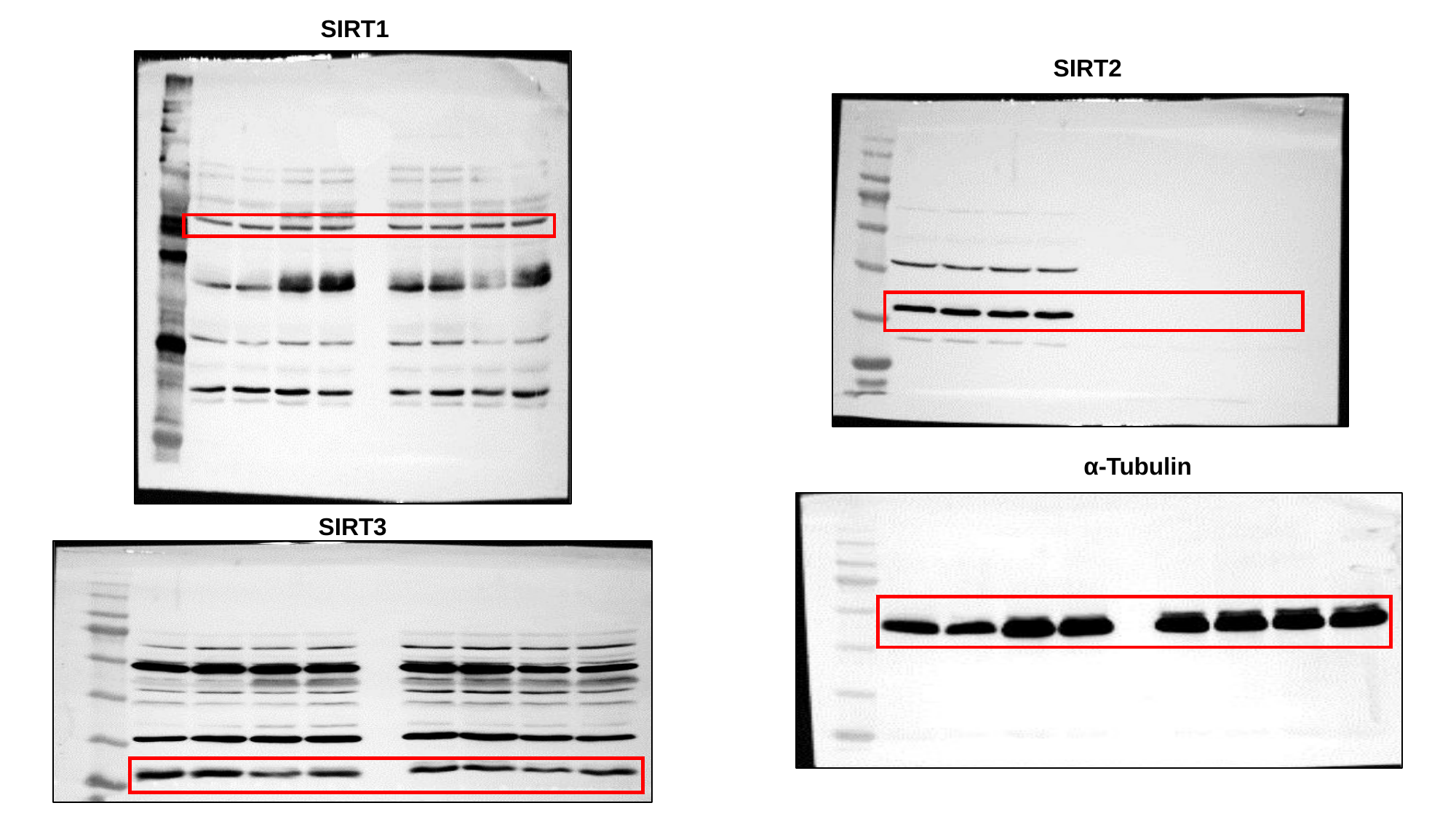

SIRT1
SIRT2
α-Tubulin
SIRT3

Supplement: Figure 4—figure supplement 1—source data 1. [file elife-85571-fig4-figsupp1-data1.pptx]

## Slide 1
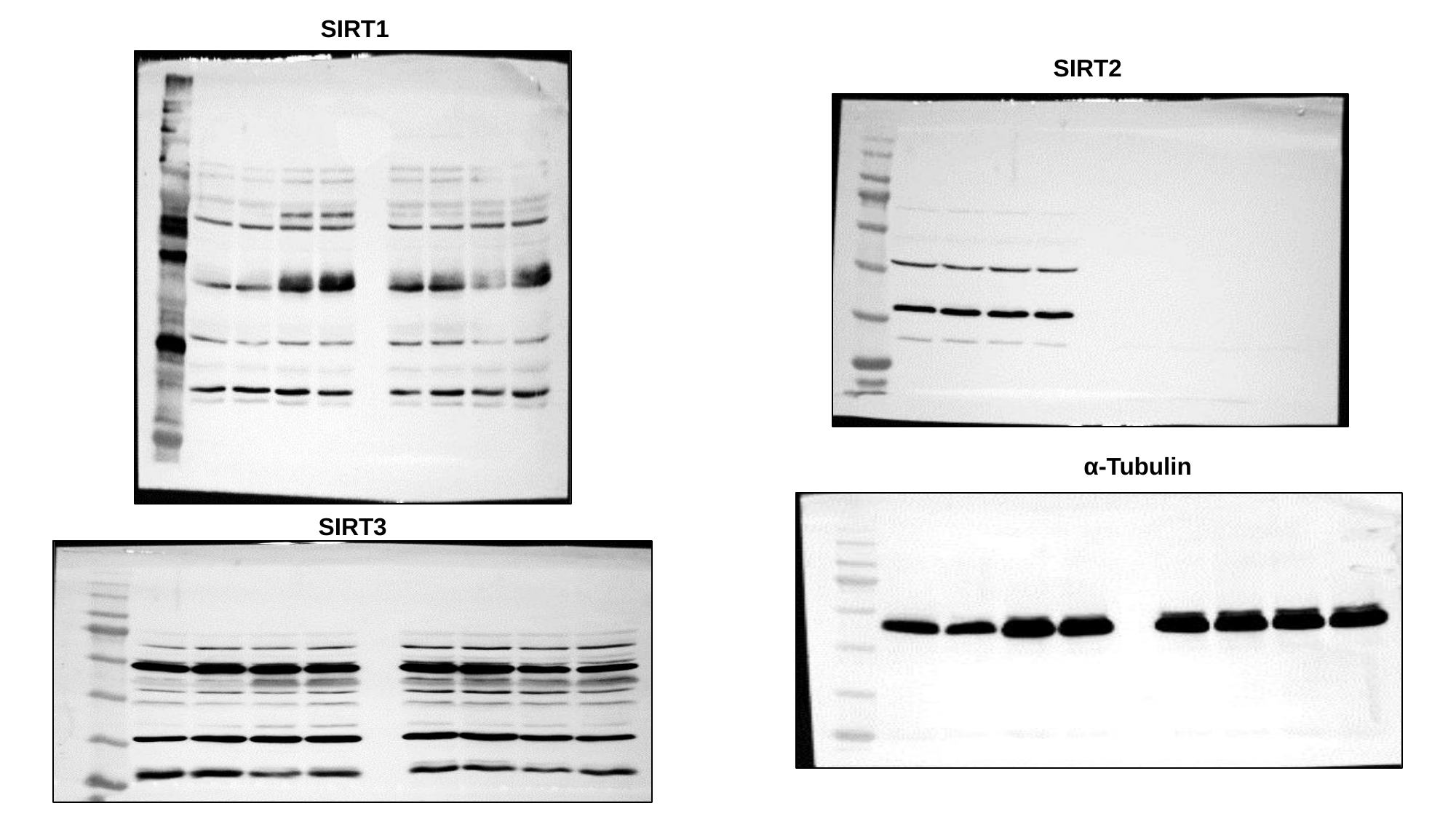

SIRT1
SIRT2
α-Tubulin
SIRT3

Supplement: Figure 4—figure supplement 1—source data 2. [file elife-85571-fig4-figsupp1-data2.pptx]

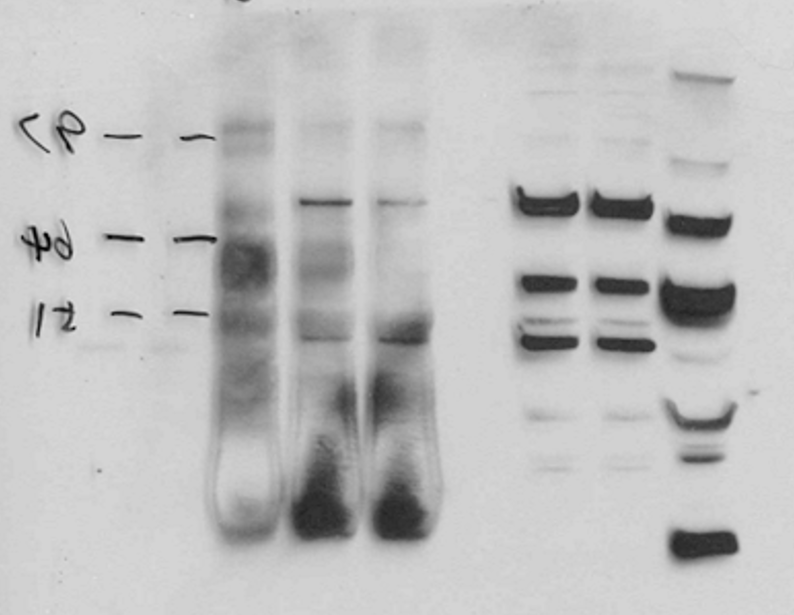

Supplement: Figure 5—source data 6. [file elife-85571-fig5-data6.zip › Figure 5A NRF2.tif]

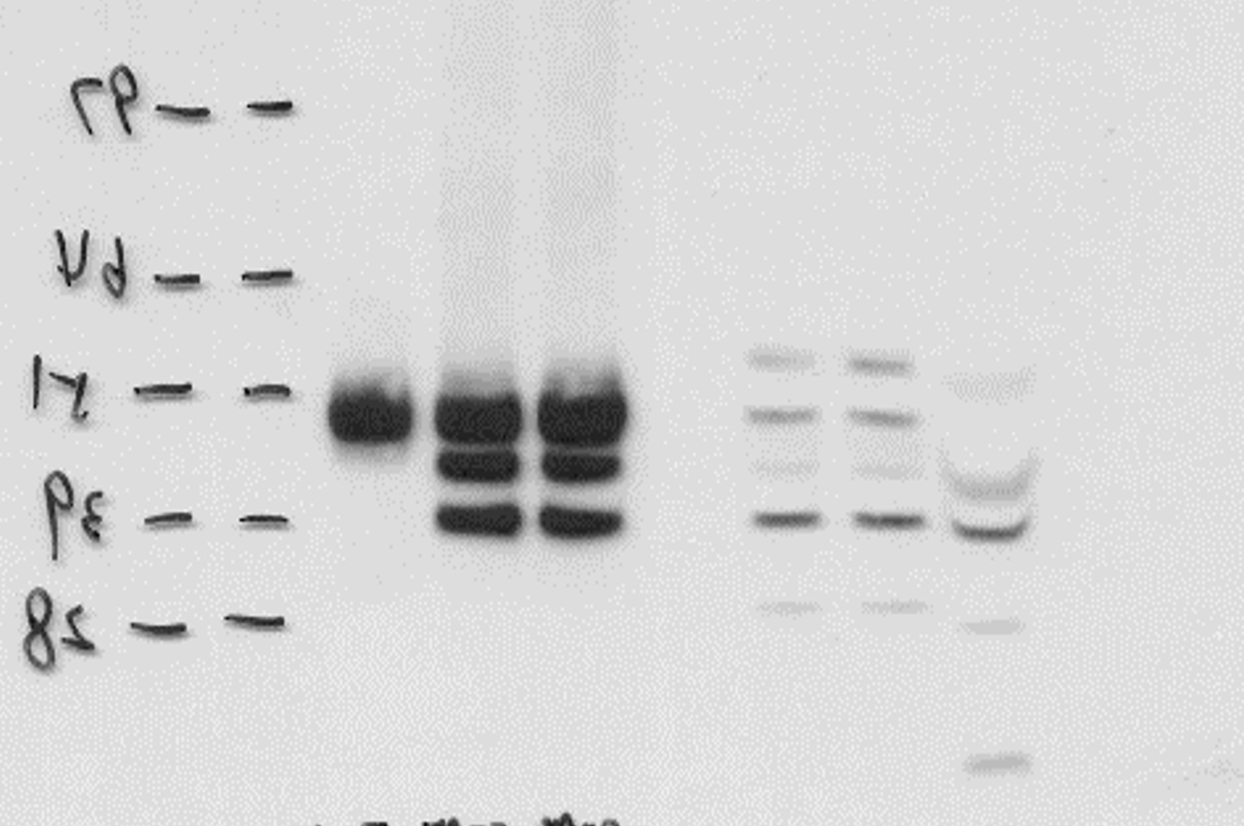

Supplement: Figure 5—source data 6. [file elife-85571-fig5-data6.zip › Figure 5A SIRT2.tif]

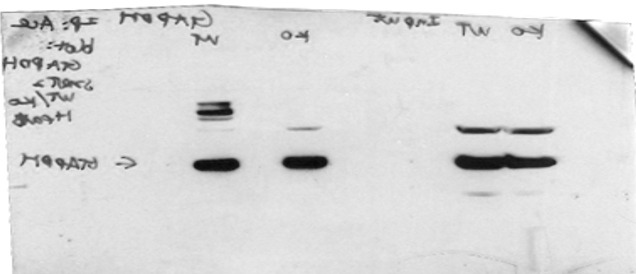

Supplement: Figure 5—source data 6. [file elife-85571-fig5-data6.zip › Figure 5B GAPDH.tif]

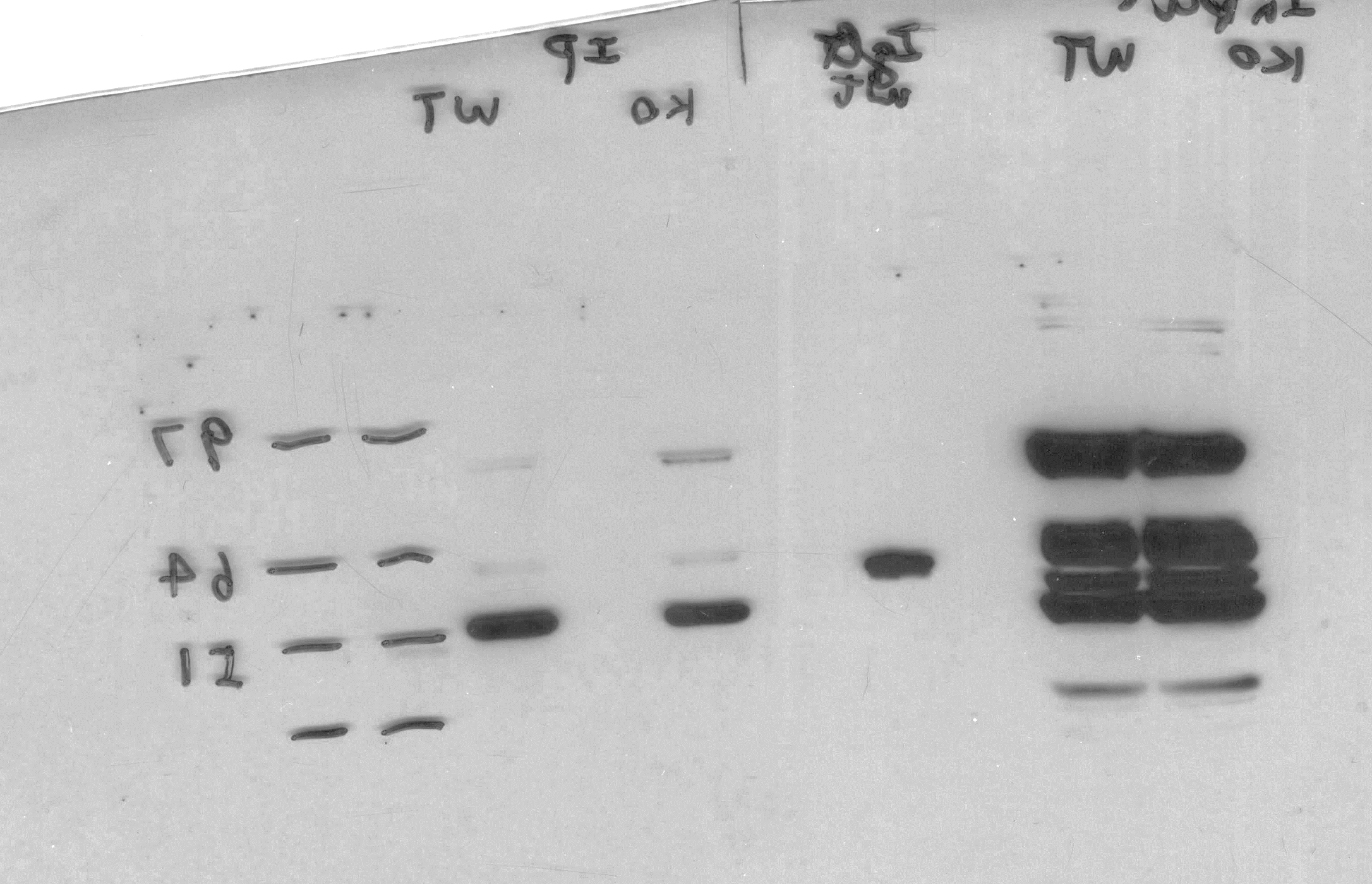

Supplement: Figure 5—source data 6. [file elife-85571-fig5-data6.zip › Figure 5B NRF2.tif]

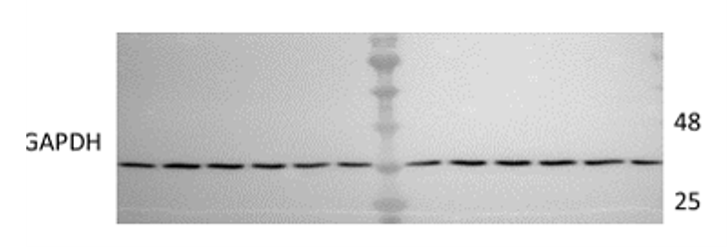

Supplement: Figure 5—source data 6. [file elife-85571-fig5-data6.zip › Figure 5C GAPDH.tif]

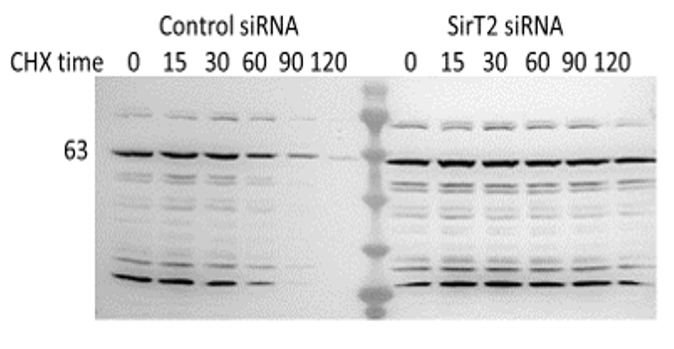

Supplement: Figure 5—source data 6. [file elife-85571-fig5-data6.zip › Figure 5C NRF2.tif]

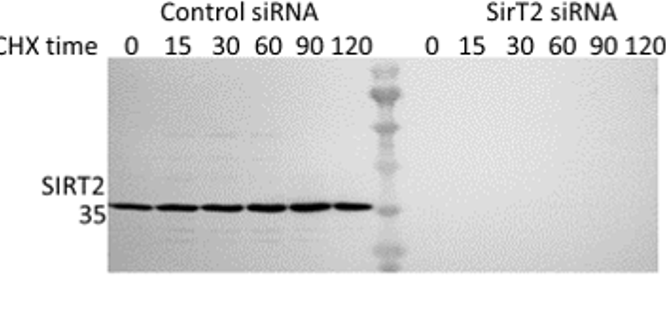

Supplement: Figure 5—source data 6. [file elife-85571-fig5-data6.zip › Figure 5C SIRT2.tif]

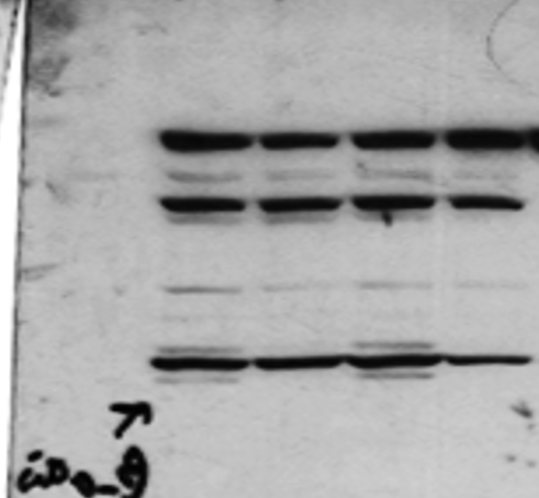

Supplement: Figure 5—source data 6. [file elife-85571-fig5-data6.zip › Figure 5D actin.tif]

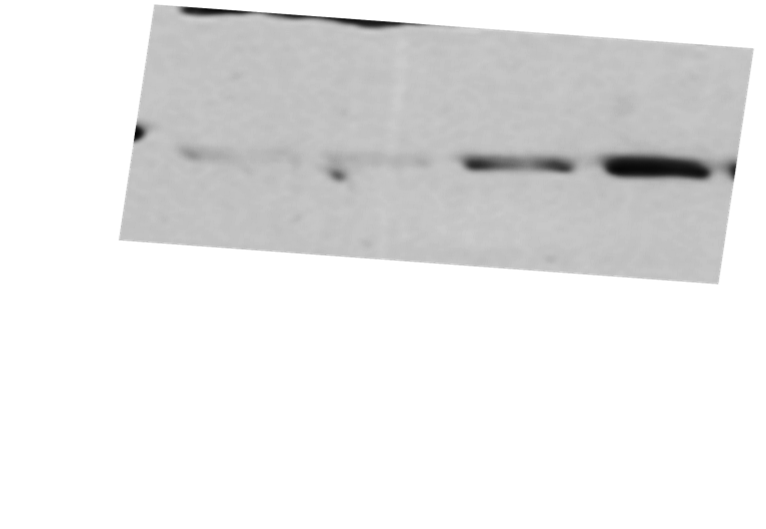

Supplement: Figure 5—source data 6. [file elife-85571-fig5-data6.zip › Figure 5D NRF2.tif]

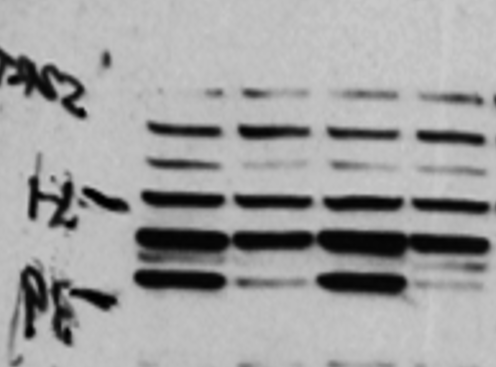

Supplement: Figure 5—source data 6. [file elife-85571-fig5-data6.zip › Figure 5D SIRT2.tif]

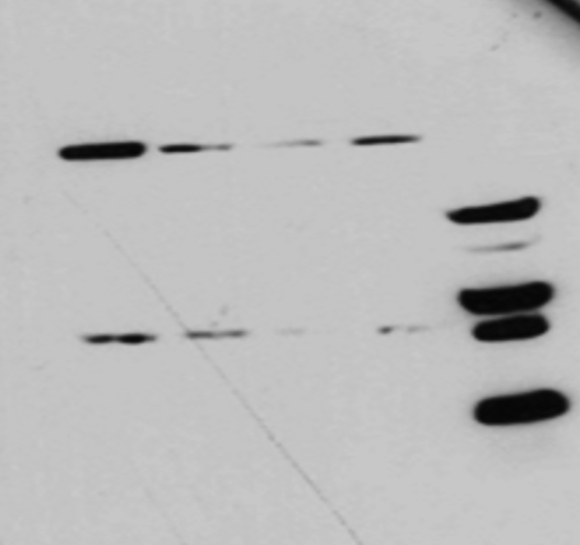

Supplement: Figure 5—source data 6. [file elife-85571-fig5-data6.zip › Figure 5E NRF2 .tif]

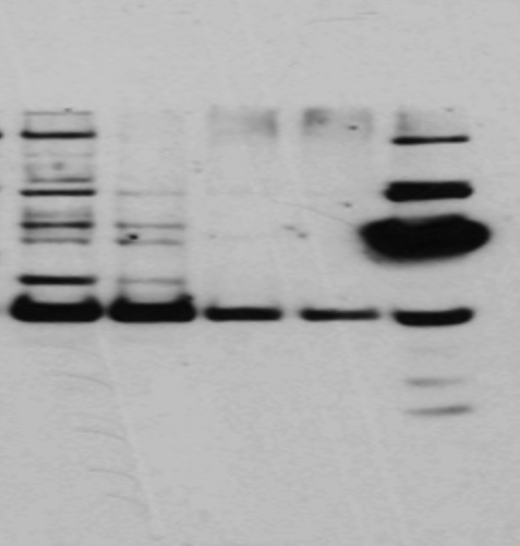

Supplement: Figure 5—source data 6. [file elife-85571-fig5-data6.zip › Figure 5E TBP .tif]

## Slide 1
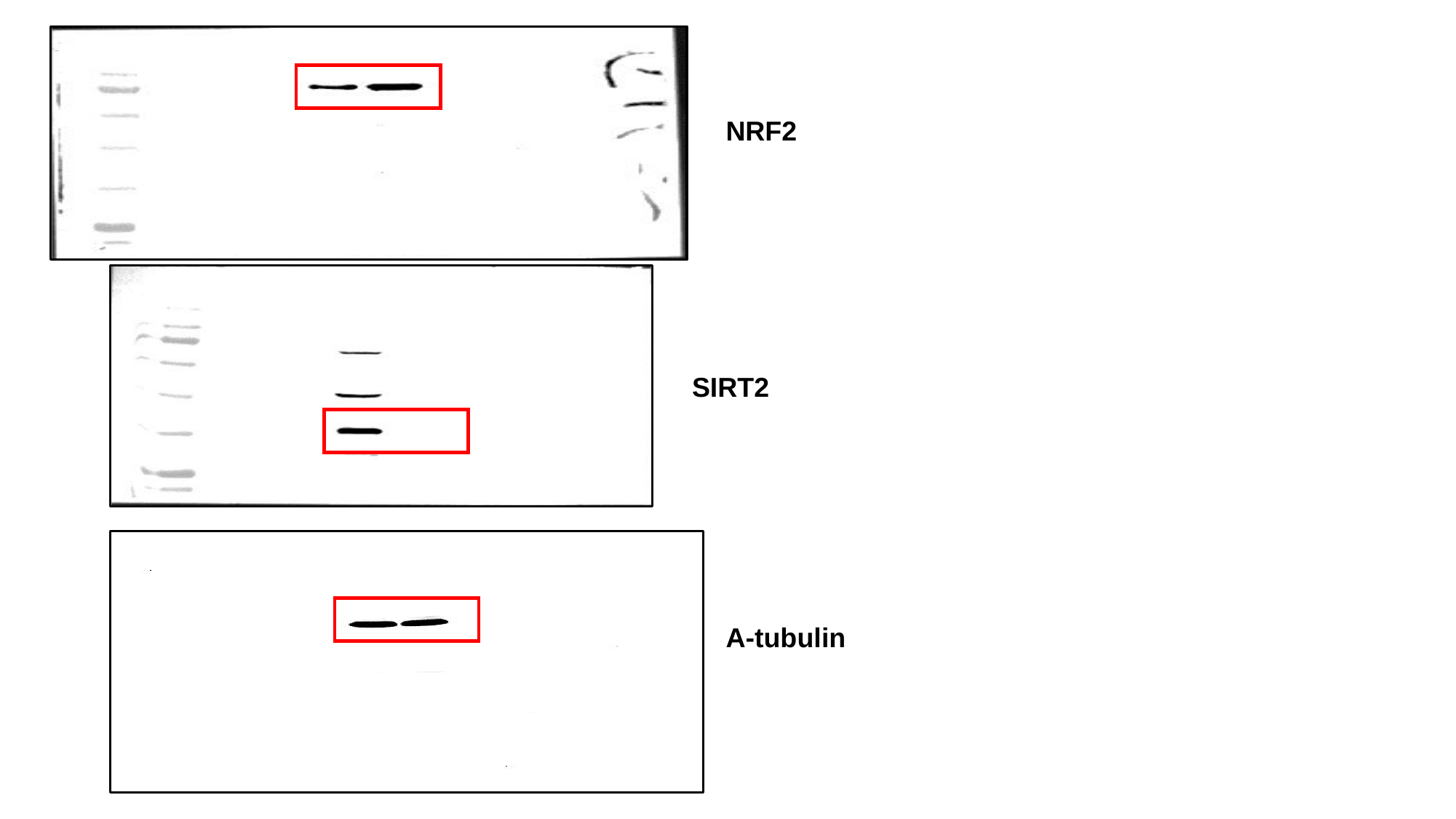

NRF2
SIRT2
Α-tubulin

Supplement: Figure 5—figure supplement 2—source data 1. [file elife-85571-fig5-figsupp2-data1.pptx]

## Slide 1
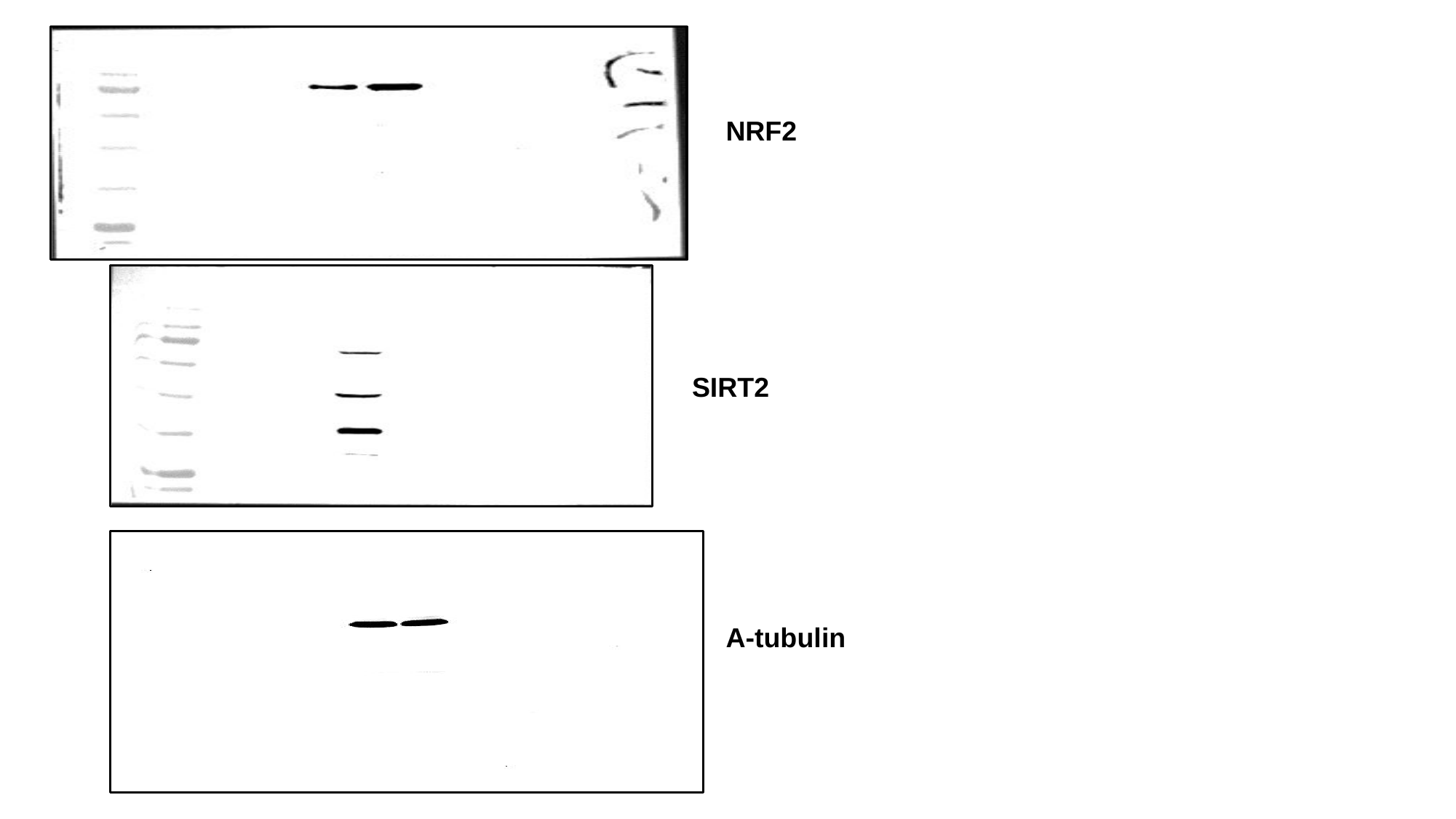

NRF2
SIRT2
Α-tubulin

Supplement: Figure 5—figure supplement 2—source data 2. [file elife-85571-fig5-figsupp2-data2.pptx]
