# Supplementary material for: SIRT2 inhibition protects against cardiac hypertrophy and ischemic injury
Source: eLife. 2023 Sep 20;12:e85571. doi: 10.7554/eLife.85571 (PMC10558204; doi:10.7554/eLife.85571)
Supplement: Figure 5—source data 5. [file elife-85571-fig5-data5.pptx]

## Slide 1
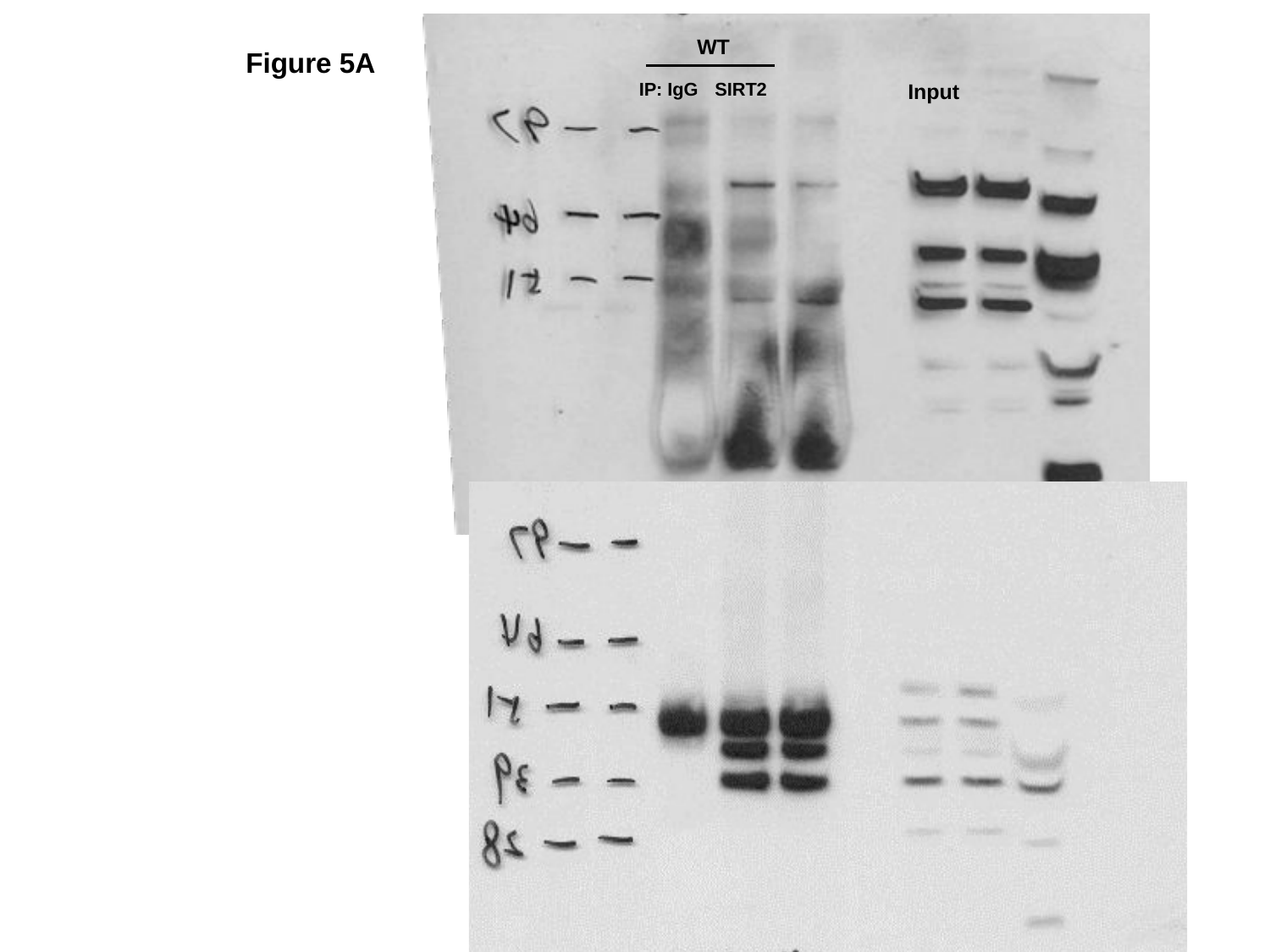

WT
SIRT2
IP: IgG
Input
Figure 5A

## Slide 2
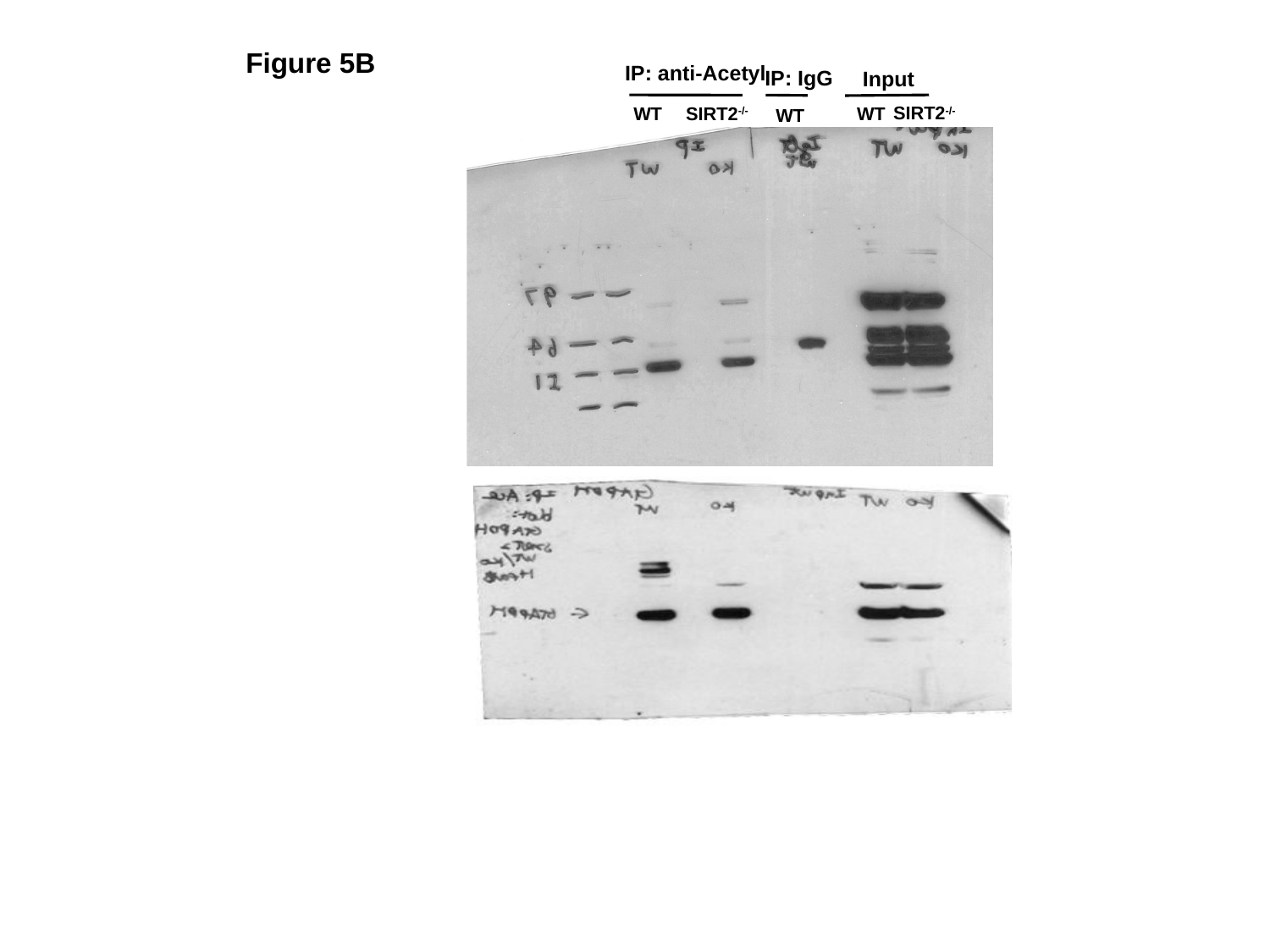

Figure 5B
IP: anti-Acetyl
IP: IgG
Input
SIRT2-/-
SIRT2-/-
WT
WT
WT

## Slide 3
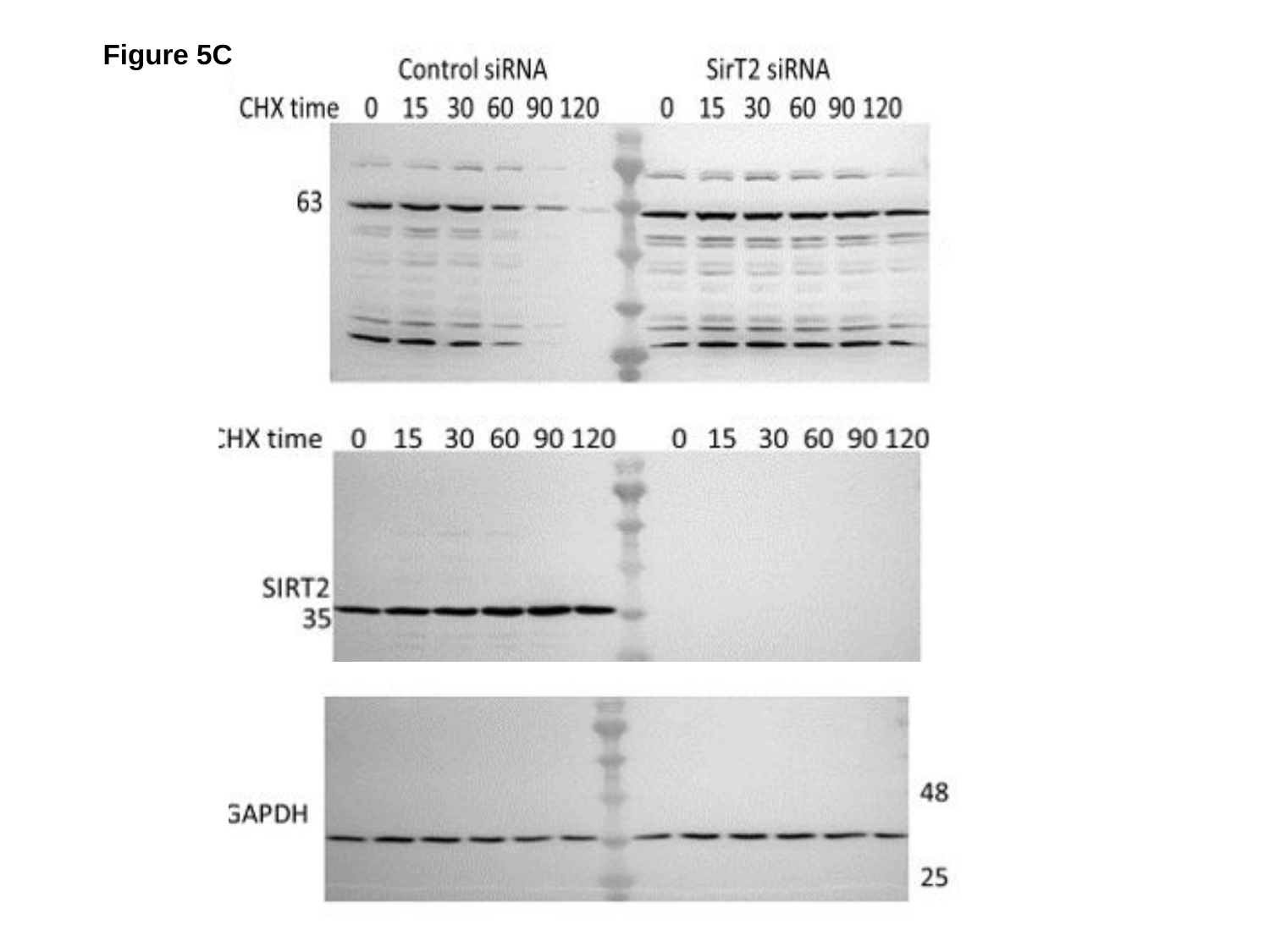

Figure 5C

## Slide 4
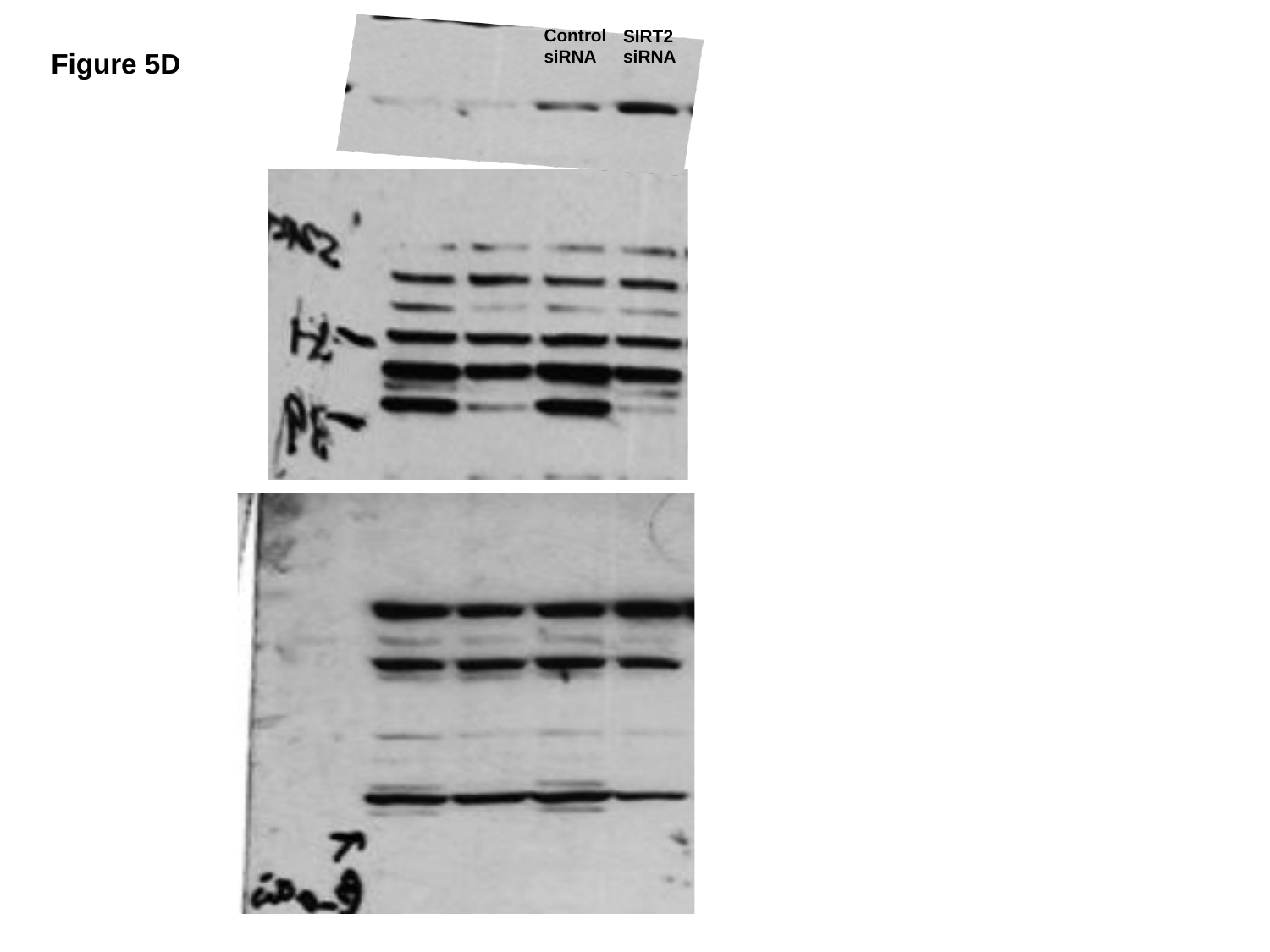

Control siRNA
SIRT2 siRNA
Figure 5D

## Slide 5
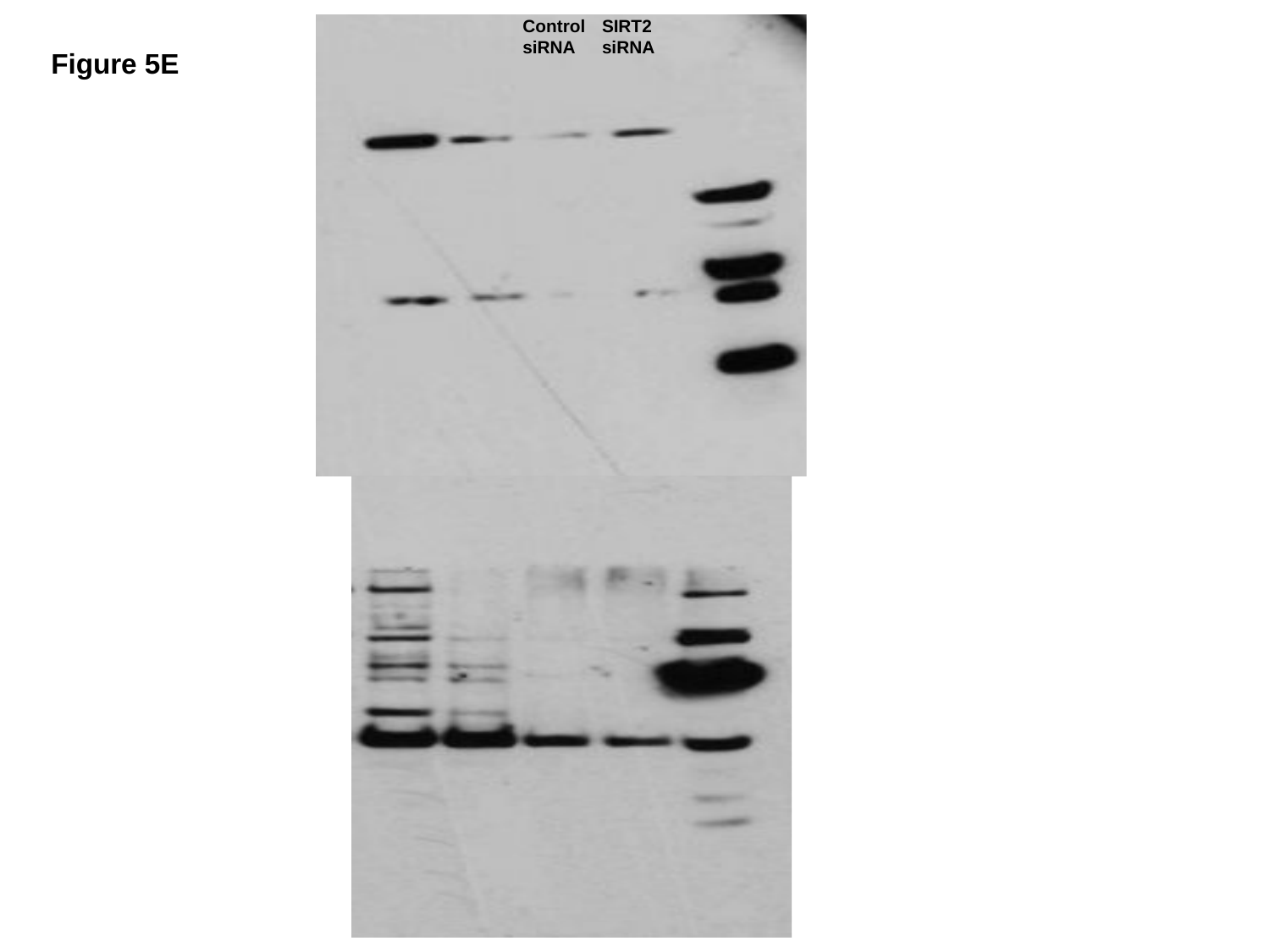

Control siRNA
SIRT2 siRNA
Figure 5E
